# Selection criteria and husbandry practices of indigenous chicken producers in Northwest Ethiopia

**DOI:** 10.1016/j.heliyon.2024.e36094

**Published:** 2024-08-10

**Authors:** Bekalu Muluneh, Mengistie Taye, Tadelle Dessie, Dessie Salilew Wondim, Damitie Kebede, Andualem Tenagne

**Affiliations:** a Department of Animal Science, College of Agriculture and Environmental Sciences, Bahir Dar University, P.O.Box 5501, Bahir Dar, Ethiopia; b Department of Animal and Range Sciences, Wolaita Sodo University, Dawuro Tarcha Campus, P.O.Box 01, Tarcha, Ethiopia; c Institute of Biotechnology, Bahir Dar University, P.O.Box 79, Bahir Dar, Ethiopia; d International Livestock Research Institute (ILRI), P.O.Box 5689, Addis Ababa, Ethiopia; e Institute of Animal Sciences, Department of Animal Breeding and Husbandry, University of Bonn, 53115, Bonn, Germany; f Department of Animal Sciences, Assosa University, P.O.Box 18, Assosa, Ethiopia

**Keywords:** Agro-ecology, Breeding objective, Chicken selection, Indigenous chicken, Trait

## Abstract

This study was conducted to identify the selection criteria and husbandry practices of chicken producers in different agro-ecological zones of Northwest Ethiopia as input for designing a breeding program. The study employed a purposive selection of districts and peasant associations with high indigenous chicken potential. The study areas were stratified based on the major agro-ecologies (highland, midland, and lowland). A total of 360 households were included in the study, and data on chicken breeding practices, selection criteria, and reproductive performance were collected and analyzed using SPSS software. In all agro-ecologies, egg production was prioritized by chicken owners when choosing female chickens. For male chickens, plumage color (index = 0.27), appearance (index = 0.24), and growth rate (index = 0.23) were the main selection factors. Farmers kept chickens primarily to generate cash through the sale of eggs and live animals (male chickens). There was a significant difference (p < 0.01) among agro-ecologies in nutritional management and housing of chickens. Chicken flock composition showed a highly significant difference (p < 0.001) among agro-ecologies, except layers. Most of the farmers had their own cock born in the flock. Chicken owners found in all agro-ecologies were practicing culling unwanted chickens. All the reproductive performance traits have shown a highly significant (p < 0.001) difference among agro-ecologies. A relatively higher inbreeding coefficient (0.18) was obtained in the highland agro-ecology compared to midland (0.16) and lowland (0.12). The study highlighted the importance of designing breeding programs that align with farmers' production objectives and trait preferences based on specific agro-ecologies for sustainable increases in chicken productivity.

## Highlights

• In addition to production traits, farmers also paid attention to morphological traits.
• Farmers raise chickens primarily for income from selling of egg and male chickens.
• Agro-ecology has a slight impact on trait preference and husbandry practices.

## 1 Introduction

Poultry production plays a crucial role in reducing poverty, ensuring food security, and generating income in developing countries [1,2]. It also provides opportunities for socio-economic improvement and inclusivity, benefiting low- and middle-income households, as well as vulnerable groups such as women, people with disabilities, orphans, and the unemployed [3,4]. Similarly, in Ethiopia, chickens hold significant socioeconomic importance, contributing to food security, income generation, religious rituals, and employment opportunities [5,6]. Rural Ethiopian families commonly raise both indigenous and exotic chicken breeds, with an estimated total poultry population of approximately 57 million, consisting of 78.85 % indigenous breeds, 9.14 % exotic breeds, and 12.03 % hybrid breeds [7].

Smallholder farmers maintain diverse flocks of indigenous chickens, meeting various economic, cultural, and religious needs [8,9]. Designing effective animal breeding programs for village chicken production requires a comprehensive understanding of farmers' management practices, production environment, breeding objectives, and trait preferences [10]. It is essential to incorporate economically significant traits into breeding programs to conserve chicken breeds, enhance productivity, and establish sustainable breeding strategies [11]. Although efforts have been made in Ethiopia to improve poultry productivity through the introduction of exotic breeds, these initiatives have faced challenges due to the intensive management requirements of such chickens [12]. Therefore, prioritizing indigenous breeds and considering the specific needs and preferences of smallholder farmers is a potential intervention strategy to enhance village poultry production.

The breeding objectives, husbandry practices, and trait preferences of chicken producers residing in diverse agro-ecological regions can vary due to differences in attitudes and environmental conditions, including area-specific farming practices. Moreover, variations in attitudes and market factors can lead to differences in these aspects between chicken owners living near urban areas and those in rural or remote locations. Several scholars have conducted studies to identify selection criteria, breeding objectives, and management practices of farmers who raise indigenous chickens in various parts of Ethiopia. However, there is a dearth of information available regarding the selection criteria and breeding objectives of farmers in the northwest region of Ethiopia, particularly in the Awi zone, West Gojjam zone, and East Gojjam zone, where a significant genetic resource of approximately 5.8 million indigenous chickens exists [7]. Furthermore, the previous studies have not addressed pocket or remote areas within the districts/zones, leaving a gap in knowledge. Therefore, the current study was set to identify the selection criteria and husbandry practices of farmers keeping indigenous chicken ecotypes found in different agro-ecologies of Northwest Ethiopia. The findings of this study will provide valuable input for the design of a sustainable breeding program tailored to the specific needs of farmers in the region.

## 2 Material and methods

### 2.1 Description of the study areas

**Fig. 1:**
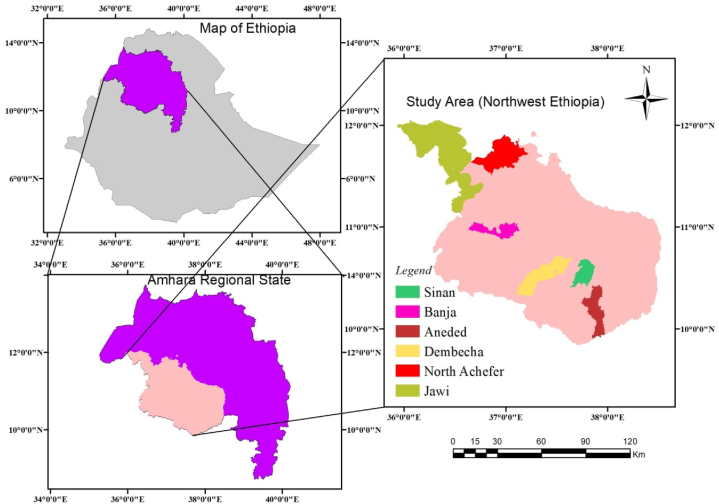
Map of the study areas.

The study was conducted in selected districts (Banja, Jawi, Sinan, Aneded, Dembecha, and North Achefer) from Amhara National Regional State, Ethiopia (Fig. 1). The agro-ecological description, the number of indigenous chickens found in the study area and the major feed resources for the chickens are presented in Table 1.

**Table 1:** Agro-ecological description, number of indigenous chickens, and major feed resources for chickens in northwest Ethiopia.

District/site	PA/*Kebele*	Agro-ecology	Altitude (m.a.s.l.)	Annual rainfall (mm)	Annual temperature (^O^C)	Indigenous Chicken number	Major feed resources for chickens
Banja	1	Highland	3028	2200–2560	7–25	36,894	wheat, maize, barley, oat, *injera*
	2	Highland	2685				
	3	Highland	2723				
Jawi	1	Lowland	995	650–1250	12–40	256,000	sorghum, finger-millet, maize, rice, *Gobe,* groundnut
	2	Lowland	1171				
	3	Lowland	1365				
Sinan	1	Highland	3214	900–1500	0–15	19,652	barley, maize, wheat
	2	Highland	3192				
	3	Highland	3081				
Aneded	1	Midland	2203	1200–1660	10–23	48,440	maize, wheat, *injera*, barley, *Engido*
	2	Midland	2071				
	3	Midland	2142				
		
Dembecha	1	Midland	1857	980–1100	18–27	113,219	wheat, maize, barley, finger-millet, *teff*
	2	Midland	2287				
	3	Midland	1979				
		
North Achefer	1	Lowland	1480	1100–1420	23–33	248,671	finger-millet, maize, sorghum, *injera*
	2	Lowland	1495				
	3	Lowland	1386				

PA= Peasant Association; m.a.s.l. = meter above sea level.

### 2.2 Sampling method

For this study, districts and peasant associations were selected purposively based on the chicken potential as per the information obtained from zonal agricultural office. The districts that are known for high indigenous chicken production and not sufficiently studied earlier were considered for the study. The districts were stratified based on major agro-ecologies (highland, midland, and lowland). Secondly, potential peasant associations (six from each agro-ecology) were selected based on information on the dissemination of exotic chickens in the past. Peasant associations, particularly found in pocket areas with no/very low distribution of exotic chickens were considered for the study. Finally, random sampling was held with the aid of agricultural extension workers among indigenous chicken owners having relatively high indigenous chicken numbers to select a total of 360 households (120 from each agro-ecology) for interview based on [13] formula for sample size determination.

### 2.3 Data collection

At each sampling site, farmers were briefed about the objective of the study before starting the data collection. A semi-structured questionnaire was designed and translated in to local language (Amharic) to address the major management practices, chicken composition, and the purpose of keeping chickens. The data was collected by trained enumerators with close supervision of investigators. The major chicken breeding practices, culling practices, and trait preferences of farmers, including their criteria to select breeding cocks and hens, were assessed. In addition, information on the effective number of breeding animals and the reproductive and production performance of chickens was collected from the chicken owners using a semi-structured questionnaire and focal group discussions with extension workers and model farmers.

### 2.4 Data management and analysis

The data was organized and analyzed using MS Excel and Statistical Package for Social Sciences (SPSS) version 26 [14]. Origin software was used to construct graph. Preliminary data analysis, such as the normality test was employed before conducting the main data analysis. Descriptive statistics, such as mean, frequency, and percentage were utilized in analyses of the data. The Pearson Chi-square test was employed to compare the qualitative variables between different agro-ecologies and Cramer's V was used to determine the magnitude of the association between variables. According to Ref. [15], a Cramer's V value greater than 0.25 indicates a strong association, while a value greater than 0.15 suggests a strong association, a value greater than 0.10 indicates a moderate association, a value greater than 0.05 implies a weak association, and a value of 0 or very close to 0 indicates no or very weak association. Mean comparisons were made using Tukey test. Indices were calculated to provide a ranking of the reasons for keeping chickens, farmers' trait preferences, and selection criteria according to the formula given by Ref. [16];

[1] $$\text{Index}=\frac{\sum \left[\left(3\times \text{rank}\;1\right)+\left(2\times \text{rank}\;2\right)+\left(1\times \text{rank}\;3\right)\right]\;\text{individual}\;\text{trait}}{\sum \left[\left(3\times \text{rank}\;1\right)+\left(2\times \text{rank}\;2\right)+\left(1\times \text{rank}\;3\right)\right]\;\text{overall}\;\text{traits}}\ldots \ldots \ldots \ldots \ldots \ldots \ldots \ldots \ldots$$

For the analysis of chicken flock composition, and chicken performance traits, the Generalized Linear Model (GLM) was used fitting agro-ecology as a fixed effect.

[2] $$Y_{ij}=\mu +A_{i\;}+\varepsilon_{ij}\ldots \ldots \ldots \ldots \ldots \ldots$$

where,

Y_ij_ = the response variables;

μ = overall mean;

A_i_ = effect of the ith agro-ecology (highland, midland, and lowland) on the respective variables;

Ɛ_ij_ = residual error term.

The coefficient of inbreeding (ΔF) was calculated from the effective number of breeding animals [17].

[3] $$\text{Ne}=\frac{4\text{Nm}\times \text{Nf}}{\text{Nm}+\text{Nf}}\ldots \ldots \ldots \ldots \ldots \ldots ‥$$

[4] $$\Delta F=\frac{1}{2\text{Ne}}\;\ldots \ldots \ldots \ldots \ldots \ldots ‥$$

where, ΔF = the inbreeding coefficient; Ne = the effective population number; Nm = number of breeding males; Nf = number of breeding females.

## 3 Results and discussions

### 3.1 Major indigenous chicken management practices

**Fig. 2:**
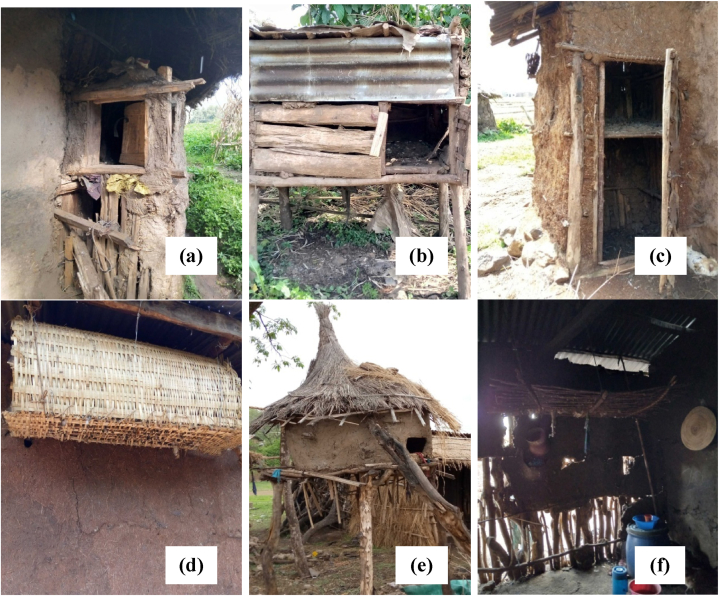
Separate chicken house in highland (a and b), midland (c and d) and lowland (e) agro-ecology; chicken shelter inside the family house in lowland agro-ecology (f).

There was a significant difference (p < 0.01) observed in the nutritional management of chickens across different agro-ecologies, as shown in Table 2. The majority of respondents (98.6 %) provided supplementary feed for their chickens. Similarly, more than 94 % of farmers in various parts of Ethiopia also offered supplementary feed to their chickens [18]. The availability of different grains resulting from mixed crop and livestock farming systems, along with farmers' desire to enhance productivity, likely contributes to the trend of supplementing chickens. Regarding housing, a significant difference (p < 0.01) was found among the agro-ecologies, possibly due to variations in farmers' perceptions and the availability of local construction materials for chicken housing. Fig. 2 illustrates distinct chicken housing structures in highland agro-ecology (a and b), midland agro-ecology (c and d), lowland agro-ecology (e), and a chicken shelter located within a family house in lowland agro-ecology (f). In the study area, a significant proportion of farmers (approximately 53.34 %) constructed separate shelters for their chickens (Table 2). This indicates their awareness of the importance of separate housing in minimizing the risk of transmissible and Zoonotic diseases. A similar finding was reported by Ref. [8], where the majority (80 %) of chicken owners in Konso had separate shelters for their chickens. In terms of vaccination, a higher proportion (71.9 %) of the respondents in the study area vaccinated their chickens, demonstrating a good understanding among farmers regarding the use of vaccination to prevent diseases. In contrast, a study by Ref. [8] revealed that about 95 % of respondents raising village chickens in Farta, Mandura, Horro, Konso, and Sheka did not immunize or vaccinate their chickens.

**Table 2:** The major management practices of indigenous chicken producers in northwest Ethiopia.

Management Practices	Agro-ecology							
	Highland N = 120		Midland N = 120		Lowland N = 120		Overall	
	N	%	N	%	N	%	N	%
**Nutritional management**								
Scavenging	0	0.00	0	0.00	5	4.2	5	1.4
Scavenging with supplement	120	100	120	100	115	95.8	355	98.6
Complete ration	0	0.00	0	0.00	0	0.00	0	0.00
*χ*^*2*^=10.14**; *Cramer's V=0.168*								
**Housing**								
In the family house	27	22.5	38	31.7	42	35	107	29.72
Separate shelter	60	50	67	55.8	65	54.2	192	53.34
Separate house with other animals	33	27.5	15	12.5	10	8.3	58	16.11
Others/Tree	0	0.00	0	0.00	3	2.5	3	0.83
*χ*^*2*^=24.93**; Cramer's V=0.186								
**Vaccination**								
Yes	92	76.7	79	65.8	88	73.3	259	71.9
No	28	23.3	41	34.2	32	26.7	101	28.1
*χ*^*2*^*=3.66*^*NS*^; *Cramer's V=0.101*								

N= Number of households; χ^2^ and *Cramer's V* values shows the association of agro-ecology with specific management practice; NS=Non-significant; **P < 0.01.

### 3.2 Composition of chicken flocks

There was a highly significant difference (p < 0.001) observed in the composition of chicken flocks among agro-ecologies, except for layers, as shown in Table 3. In contrast to the findings of this study [19], reported that the average number of chicken flocks per household did not differ between lowland and highland agro-ecologies in different regions of Ethiopia. In highland and midland agro-ecologies, a larger number of chicks and pullets were found in addition to layers. The higher number of layers suggests that farmers primarily keep indigenous chickens for egg production. However, in the lowland agro-ecology, a higher proportion of chicks (34.9 %) per household were recorded compared to layers (23.2 %) and pullets (19.7 %). Similarly, a higher number of young chicks, followed by hens, were reported in the Burie district of Northwest Ethiopia [20] and the Indian Himalayan region [21]. The higher proportion of layers and chicks in the study area may be attributed to the farmers' aim of producing a larger number of eggs for sale and hatching for stock replacement. The lower proportion of cocks, particularly in the highland agro-ecology, could be due to selling cockerels and cocks for income generation and/or utilizing neighboring cocks for breeding purposes. This lower proportion of cocks and cockerels in the area may lead to increased inbreeding and the production of more unfertile eggs. The significant difference in chicken flock composition among different agro-ecologies may be influenced by variations in the breeding objectives of farmers who keep the chicken flocks.

**Table 3:** Average chicken flock holding per household in different agro-ecologies of northwest Ethiopia.

Chicken flock	Agro-ecology								p-value
	Highland (N = 120)		Midland (N = 120		Lowland (N = 120		Overall		
	Mean ± SD %		Mean ± SD %		Mean ± SD %		Mean ± SD %		
Layers	4.38 ± 2.0	43.5	4.8 ± 2.6	41	4.18 ± 2.74	23.2	4.45 ± 2.47	33.5	NS
Cocks	0.82 ± 0.89^b^	8.1	1.24 ± 0.93^a^	10.6	1.44 ± 0.99^a^	7.99	1.17 ± 0.97	8.8	***
Pullets	1.29 ± 2.04^c^	12.8	2.24 ± 2.09^b^	19.2	3.55 ± 2.47^a^	19.7	2.36 ± 2.39	17.8	***
Cockerel	0.64 ± 1.15^b^	6.4	1.12 ± 1.71^b^	9.5	2.58 ± 2.46^a^	14.3	1.44 ± 2.02	10.9	***
Chicks	2.94 ± 4.37^b^	29.2	2.31 ± 3.59^b^	19.7	6.3 ± 6.68^a^	34.9	3.85 ± 5.34	29	***

N= Number of households; SD=Standard deviation; NS=Non-significant; ^a,b,c^Means across a row with different superscript letters denote significant differences at P < 0.001; Flock: a group of chicken with different age and sex categories.

### 3.3 Purpose of keeping chickens

The table presented, Table 4, outlines the ranking of chicken production objectives as perceived by smallholder farmers. Across all agro-ecologies, the main reasons for keeping male chickens were income generation (0.36), breeding for replacement stock (0.30), and meat consumption (0.21). These findings align with the results of [20], who reported that the primary purposes of chicken rearing in the Burie district were income generation (51 %), breeding (45 %), and home consumption (44 %). In the highland and midland agro-ecologies, the primary reason for keeping female chickens was egg production for selling, with index values of 0.41 and 0.39, respectively. Similarly, in various agro-ecologies of Ethiopia, indigenous chickens were primarily kept for income generation through the sale of eggs [18]. In the lowland agro-ecology, the main purposes of keeping chickens were egg production for home consumption (0.27) and selling (0.26), followed by breeding (0.25). These findings consistent with [22], who reported that indigenous chicken rearing in the West Oromia region of Ethiopia primarily focused on egg consumption and egg selling. The variation in chicken functions among different agro-ecologies can be attributed to socio-economic, socio-cultural, and perception differences among chicken owners. For example, in some areas studied, there was no tradition of consuming eggs, and even during the fasting season, eggs intended for hatching purposes were observed to rot instead of being provided to babies.

**Table 4:** Farmers' purpose of keeping male and female chickens in northwest Ethiopia.

Purpose	Agro-ecology												
	Highland (N = 120)				Midland (N = 120)				Lowland (N = 120)				Overall index
	Rank			Index	Rank			Index	Rank			Index	
	1st	2nd	3rd		1st	2nd	3rd		1st	2nd	3rd		
**Male chickens**													
Income (animal sale)	53	56	8	0.39	50	55	5	0.37	35	59	15	0.33	0.36
Breeding	50	26	23	0.31	61	15	14	0.32	39	17	47	0.28	0.30
Meat(home consumption)	14	31	37	0.20	6	26	42	0.16	40	28	23	0.28	0.21
Saving	3	6	38	0.08	3	14	21	0.07	2	6	25	0.06	0.07
Manure	0	0	4	0.01	0	1	19	0.03	0	0	6	0.01	0.02
Ceremony	0	1	10	0.01	1	8	18	0.05	4	10	4	0.05	0.04
**Female chickens**													
Income (Egg sale)	77	24	17	0.41	72	29	10	0.39	38	28	16	0.26	0.35
Egg (home consumption)	27	52	14	0.28	21	41	21	0.24	26	51	17	0.27	0.26
Income (animal sale)	4	27	55	0.17	8	36	30	0.18	7	18	48	0.14	0.16
Breeding	10	13	26	0.11	14	11	23	0.12	48	5	29	0.25	0.16
Meat (home)	0	0	5	0.01	5	2	12	0.04	1	14	5	0.05	0.03
Saving	2	1	3	0.02	0	1	14	0.02	0	0	5	0.01	0.02
Manure	0	3	1	0.01	0	0	6	0.01	0	0	0	0.00	0.01
Ceremony	0	0	0	0.00	0	0	4	0.01	3	4	0	0.02	0.01

N= Number of households or respondents.

### 3.4 Selection criteria for breeding cocks and hens

The selection criteria for breeding cocks varied among different agro-ecologies, with the highland agro-ecology prioritizing growth rate/body weight (0.29), appearance (0.28), and plumage color (0.24), as indicated in Table 5. In the midland agro-ecology, comb type (0.26), plumage color (0.23), and growth rate (0.21) were considered the most important traits for selecting breeding cocks. In the lowland agro-ecology, plumage color (0.33), appearance (0.25), and comb type (0.17) were the major criteria for selection. Consistent with the current study, farmers in the Amhara (Farta) and Oromia (Horro) regions also emphasized plumage color as the primary selection criterion [8]. However, farmers in Kenya [23] and Rwanda [24] did not prioritize plumage color in their selection of chickens, which contrasts with the findings of this study. The preference for morphological traits such as plumage color and comb type over growth and adaptive traits (disease resistance, mothering ability, and scavenging ability) in the current study area can be attributed to socio-cultural factors, where farmers place importance on the visual aesthetics of chickens. For instance, red plumage and a double comb are favored over other traits for male chickens in most surveyed areas, and these traits can significantly influence the market price of chickens.

**Table 5:** Ranking of selection criteria for breeding cocks and hens in northwest Ethiopia.

Class and section criteria	Agro-ecology												
	Highland (N = 120)				Midland (N = 120)				Lowland (N = 120)				Overall index
	Rank			Index	Rank			Index	Rank			Index	
	1st	2nd	3rd		1st	2nd	3rd		1st	2nd	3rd		
**Breeding cocks**													
Plumage color	21	34	39	0.24	33	23	18	0.23	53	22	31	0.33	0.27
Appearance	35	31	33	0.28	13	31	26	0.18	16	44	45	0.25	0.24
Growth rate	50	24	13	0.29	27	19	31	0.21	23	24	16	0.18	0.23
Comb type	14	21	30	0.16	36	24	31	0.26	26	26	19	0.21	0.21
Disease resistance	0	8	3	0.03	1	15	7	0.06	0	4	5	0.02	0.04
Scavenging ability	0	1	2	0.01	2	6	6	0.03	0	0	4	0.01	0.02
Longevity	0	2	0	0.01	8	1	3	0.04	2	0	0	0.01	0.02
**Breeding hens**													
Egg number	56	26	22	0.34	74	5	15	0.34	22	26	30	0.20	0.29
Appearance	30	17	20	0.20	6	18	13	0.09	32	19	18	0.21	0.17
Plumage color	11	20	15	0.12	5	24	16	0.11	32	15	23	0.21	0.15
Broodiness	1	7	10	0.04	9	18	22	0.12	12	28	10	0.14	0.10
Egg fertility	9	17	6	0.09	15	19	11	0.13	8	14	6	0.08	0.10
Comb type	11	17	18	0.12	11	9	13	0.09	1	9	20	0.06	0.09
Mothering ability	0	8	9	0.03	0	15	20	0.07	13	4	4	0.07	0.06
Disease resistance	1	5	13	0.04	0	9	4	0.03	0	5	2	0.02	0.03
Longevity	1	2	5	0.02	0	3	6	0.02	0	0	6	0.01	0.02
Scavenging ability	0	1	3	0.01	0	0	0	0.00	0	0	1	0.001	0.00

N= Number of households or respondents.

In terms of selecting breeding hens, the most important traits were egg number (0.29), appearance (0.17), and plumage color (0.15), as shown in Table 5. This aligns with the findings of [22] in the BakoTibe district of the Western Oromia region, where respondents utilized egg production as a key criterion for selecting and retaining hens in the flock. The selection of chickens based on egg production traits indicates that the primary breeding objective of farmers in the study areas is focused on egg production. In the highland agro-ecology, the most important traits for selecting breeding hens were egg number (0.34), appearance (0.20), comb type (0.12), and plumage color (0.12). In the midland agro-ecology, egg number (0.34), egg fertility (0.13), and broodiness (0.12) were the primary considerations. Farmers in the midland agro-ecology preferred hens with low brooding frequency to maximize egg production. In the lowland agro-ecology, plumage color (0.21), appearance (0.21), and egg number (0.20) were the most important traits for selecting breeding hens. In contrast to the current study [10], reported that farmers in the Gurage zone of Ethiopia prioritized adaptive traits (disease and scavenging ability) over production traits in all agro-ecologies. Similarly, a study conducted in Kenya found that egg yield, mothering ability, and body size were the most preferred traits among chicken farmers [25]. Studies conducted in the Indian Himalayan region [21] revealed that most households chose to rear indigenous chickens due to their adaptability and survivability. This preference suggests that farmers, especially those in the sub-temperate agro-ecology of the Himalayan region, prioritize these traits when selecting indigenous chickens, considering the challenging climatic conditions. Ethiopia's indigenous chicken breeds are known for their mothering ability, disease resistance, and the taste of their meat and eggs [26]. The lack of emphasis on adaptive traits in the current study area might be due to the presence of these unique characteristics in the indigenous chickens, which directs farmers' focus towards other traits. Considering farmers' preferences for specific traits is vital in designing sustainable improvement programs [27]. Failure to incorporate stakeholders' needs in breeding programs can lead to rejection by end users, as highlighted by Ref. [28].

**Fig. 3:**
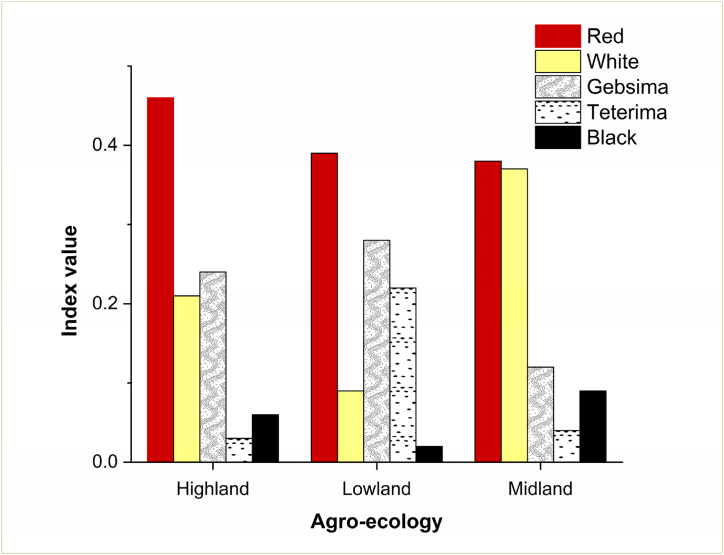
Ranking index value for plumage color preference of farmers keeping indigenous chicken in different agro-ecologies of northwest Ethiopia. *Gebsima* = wheaten strips on a black background; *Teterima* = black spot on white.

Fig. 3 shows the plumage color preferences of chicken owners. Across all agro-ecologies, the most favored color types were red, white, and *Gebisma* (wheaten strips on a black background, grayish with varying mixture, and red brownish with black). However, in the lowland agro-ecology, *Teterima* (black spot on white, black with white tips, and white with black or red spots) were more preferred than white. Likewise, the chicken owners in Benshangul-Gumuz (Mandura), Oromia (Horro), and Southern Regions (Konso and Sheka) preferred red plumage color, while farmers in Amhara (Farta) favored white plumage color, as noted by Ref. [8]. On the other study, farmers in Mezhenger, Sheka, and Benchi-Maji zones of southwestern Ethiopia predominantly preferred red plumage color [29]. However, the same study also reported black as the second most preferred color for selecting cocks, which contradicts the findings of the current study. In the present study, black plumage color was not preferred by farmers in any agro-ecology due to its cultural significance. Farmers generally avoid slaughtering chickens with black plumage color, especially during holidays, and these chickens tend to have lower market prices compared to others. In some areas, farmers refrain from choosing chickens with white plumage color because they are more visible and can be easily targeted by predators. Furthermore, some chicken owners hold the belief that white plumage color is attractive and that the "evil eye" of people could affect the growth and production of the chickens. These factors contribute to the dominance of red plumage color across all agro-ecologies.

### 3.5 Breeding and culling practices

A highly significant difference (p < 0.001) was observed across different agro-ecologies in terms of brood modification methods. In the highland and midland agro-ecologies, the majority of farmers (31.7 % and 56.7 %, respectively) practiced hanging their broody hen's upside-down to modify brooding (Table 6). This aligns with the findings of [29] in Mezhenger, Sheka, and Benchi-Maji zones of southwestern Ethiopia, where 38.2 % of chicken owners employed the upside-down hanging method for brood modification. In contrast, most lowland chicken owners in the current study (43.3 %) opted to change the brooding place as their preferred method. The variation in brood modification methods among agro-ecologies could be attributed to differences in farmers' perceptions. Some farmers in the study area consider hanging chickens for brooding modification as unethical, and there is a decreasing trend in the use of this method over time. The study revealed that almost all farmers practiced an uncontrolled (natural) mating system. Out of the households surveyed, 76.9 % owned their own breeding cock, and the majority (70 %) of these cocks was obtained from their own flock. While owning a breeding cock can positively impact the fertility of eggs from hens, utilizing cocks from their own flock can increase the risk of inbreeding. Similar findings were reported in the Burie district of Northwest Ethiopia, where 70.7 % of chicken owners had their own cocks, primarily sourced from their own flocks [20].

**Table 6:** Breeding and culling practices of indigenous chicken producer farmers in northwest Ethiopia.

Breeding Practices	Agro-ecology							
	Highland (N = 120)		Midland (N = 120)		Lowland (N = 120)		Overall	
	N	%	N	%	N	%	N	%
**Brood modification**								
Hanging upside-down	38	31.7	68	56.7	12	10	118	32.78
Change brooding place (In house)	36	30	27	22.5	52	43.3	115	31.94
Moving to neighbor houses	35	29.2	22	18.3	46	38.3	103	28.61
Do nothing	4	3.3	2	1.7	10	8.3	16	4.44
Submerge into water up to the breast	6	5	1	0.8	0	0.00	7	1.94
Other/tie wings separately	1	0.8	0	0.00	0	0.00	1	0.28
*χ*^*2*^=74.06***; *Cramer's V=0.321*								
**Own cock**								
Yes	67	55.8	103	85.8	107	89.2	277	76.9
No	53	44.2	17	14.2	13	10.8	83	23.1
*χ*^*2*^=45.597***; *Cramer's V=0.356*								
**Sources of breeding cock**								
Own (private flock)	42	35	74	61.7	78	65	194	70
Purchased (Market)	25	20.8	29	24.2	29	24.2	83	30
*χ*^*2*^*=2.302*^*NS*^; *Cramer's V=0.091*								
**If no cock, how do you breed your hens**								
From neighbor	49	40.8	17	14.2	13	10.8	79	95.2
I do not need a cock for my hens	4	3.3	0	0.00	0	0.00	4	4.8
*χ*^*2*^*=2.379*^*NS*^; *Cramer's V=0.169*								
**Culling criteria**								
Old age	44	36.7	41	34.2	45	37.5	44	36.1
Poor productivity	46	38.3	40	33.3	43	35.8	43	35.8
Sickness	20	16.7	25	20.8	19	15.8	21	17.8
Lack of broodiness	10	8.3	14	11.7	13	10.8	12	10.3
*χ*^*2*^*=2.29*^*NS*^; *Cramer's V=0.891*								
**Culling method**								
Home consumption	35	29.2	41	34.2	39	32.5	115	31.9
Sale	83	69.2	74	61.7	79	65.8	236	65.6
Sacrifice/cultural purpose	2	1.7	5	4.2	2	1.7	9	2.5
*χ*^*2*^*=3.004*^*NS*^; *Cramer's V=0.065*								
**Culling age (male)**								
≥2 years	39	35.5	29	24.2	46	38.3	114	31.7
≥3 years	68	56.7	58	48.3	52	43.3	178	49.4
≥4 years	12	10	14	11.7	16	13.3	42	11.7
≥5 years	1	8	19	15.8	6	5	26	7.2
*χ*^*2*^=43.584***; *Cramer's V=0.246*								
**Culling age (female)**								
≥2 years	30	25	29	24.2	46	38.3	105	29.2
≥3 years	73	60.8	58	48.3	52	43.3	183	50.8
≥4 years	15	12.5	14	11.7	16	13.3	45	12.5
≥5 years	2	1.7	19	15.8	6	5	27	7.5
*χ*^*2*^=26.725***; Cramer's V=0.193								

N=Number of households; χ^2^ and *Cramer's V* values shows the association of agro-ecology with specific breeding practice; NS=Non-significant; ***P < 0.001.

All chicken owners in the study practiced culling unwanted chickens from their flocks, primarily due to old age (36.1 %), poor productivity (35.8 %), sickness (17.8 %), or lack of broodiness (10.3 %). A similar study conducted in different agro-ecologies of Ethiopia reported that the majority (91 %) of farmers practiced culling their chickens due to old age, sickness, low production, and brooding frequency [18]. The majority of respondents culled male (49.4 %) and female (50.8 %) chickens when they reached more than three years of age, as indicated in Table 6. This corresponds with the overall mean culling age of 3.37 ± 1.24 years found in southwestern Ethiopia [29]. In contrast, larger (4.3 years) and smaller (2.7 years) average culling ages were reported in Southern Ethiopia [30] and Northwest Ethiopia [20], respectively. The variation in culling age among different areas reflects the differing purposes and functions of chickens for farmers. Some farmers may choose to cull chickens earlier than expected for income generation purposes. The primary purposes for culling chickens among chicken owners were selling (65.6 %) and home consumption (31.9 %). This aligns with previous studies conducted in the Haramaya district of Eastern Ethiopia [31], where selling and home consumption were identified as the major reasons for culling.

### 3.6 Reproductive and production performance of indigenous chickens

All the reproductive and production performance traits showed a highly significant difference (p < 0.001) among the agro-ecologies (Table 7). The average age at first mating for hens was 5.07 ± 0.74 months in the highland area, 4.93 ± 0.65 months in the midland area, and 5.39 ± 1.22 months in the lowland area. These findings are consistent with [32], who reported an average age of 5.2 months for indigenous pullets at first mating in Northwest Ethiopia. Other studies also reported ages at first mating of 6.51 months in Horro district [33] and 26.15 weeks in BakoTibe and Dano districts [22]. The estimated average age for male chickens to reach sexual maturity was 4.81 ± 0.79 months in the highland area, 4.86 ± 0.78 months in the midland area, and 5.31 ± 1.11 months in the lowland area (Table 7). In comparison [29], reported an overall mean age of 4.9 months for cocks at first mating in southwestern Ethiopia. The observed differences in the age of sexual maturity for hens and cocks among the agro-ecologies could be attributed to genetic and environmental factors. In this study, the average age for chickens to lay their first egg was 5.61 ± 0.93 months. This is shorter than the findings of [18], who reported an average of 6.54 ± 0.063 months for pullets to lay their first egg in different agro-ecologies of Ethiopia.

**Table 7:** The mean reproductive and production performance of indigenous chickens as estimated by farmers among different agro-ecologies in northwest Ethiopia.

Parameters	Agro-ecology				p-value
	Highland N = 120	Midland N = 120	Lowland N = 120	Overall N = 360	
	Mean ± SD	Mean ± SD	Mean ± SD	Mean ± SD	
AFM (months, female)	5.07 ± 0.74^b^	4.93 ± 0.65^b^	5.39 ± 1.22^a^	5.13 ± 0.93	***
AFM (months, male)	4.81 ± 0.79^b^	4.86 ± 0.78^b^	5.31 ± 1.11^a^	4.99 ± 0.93	***
Age at first egg (months)	5.49 ± 0.73^b^	5.38 ± 0.69^b^	5.95 ± 1.12^a^	5.61 ± 0.93	***
Eggs per hen per clutch	16.48 ± 3.13^a^	15.38 ± 4.02^b^	14.1 ± 2.61^c^	14.99 ± 3.59	***
Eggs per hen per year	83.39 ± 32.29^a^	82.27 ± 23.37^a^	64.36 ± 15.9^b^	76.67 ± 26.2	***

N=Number of households; AFM = Age at 1^st^mating; SD=Standard deviation; ^a,b,c^Means across a row with different superscript letters denote significant differences at P < 0.001.

The highland agro-ecology exhibited a higher number of eggs per hen per clutch (16.48 ± 3.13) compared to other agro-ecologies, with an overall value of 14.99 ± 3.59 eggs. This is higher than the reported value of 13.38 eggs per hen per clutch in the Western Oromia region of Ethiopia [22]. According to the respondents, the total number of eggs per hen per year was higher in the highland agro-ecology (83.4 ± 32.29) compared to the midland and lowland areas (82.27 ± 23.37 and 64.36 ± 15.9, respectively), as shown in Table 7. In contrast [18], reported that the midland agro-ecology had a higher number of eggs compared to other agro-ecologies, with a total annual egg production of 61.89 eggs. A study conducted by Ref. [29] reported a relatively lower number of eggs per hen per year (54.7) in southwestern Ethiopia. The improved performance of chickens in the current study compared to previous studies could be attributed to better management and breeding practices by farmers, as well as genetic variations among indigenous chickens.

### 3.7 Effective population size and level of inbreeding

The number of breeding male and female chickens, the effective population size, and the rate of inbreeding of indigenous chickens in the study area are presented in Table 8. The lowland agro-ecology exhibited a lower inbreeding coefficient (0.12) compared to other agro-ecologies. In contrast, the highland agro-ecology showed a higher coefficient of inbreeding (0.18). The higher inbreeding coefficient in the highland agro-ecology could be attributed to the presence of a smaller number of chickens within households. Increasing the number of chickens would result in a larger effective population size, as it would increase the likelihood of having a greater number of breeding animals. This finding differs from a study conducted in the highland agro-ecology of the Gurage zone, which reported a smaller inbreeding coefficient (0.06) compared to other agro-ecologies [10]. However, the present study consistent with the findings of [18] for the midland agro-ecology, which reported a 12.8 % inbreeding coefficient. Nevertheless, the inbreeding coefficients in the highland (7 %) and lowland (11.3 %) agro-ecologies were lower than those found in the current study. Another study by Ref. [8] reported inbreeding coefficients of 0.096, 0.10, 0.12, 0.144, and 0.157 for Konso, Horro, Mandura, Farta, and Sheka chickens, respectively. The significant variation in inbreeding coefficient values among agro-ecologies and different studies may be attributed to variations in estimation methods and breeding practices employed by farmers. Factors such as the absence of a breeding male within the flock, uncontrolled mating, limited awareness about inbreeding, and small flock sizes can contribute to the accumulation of inbreeding and a decrease in genetic diversity [17]. The inbreeding coefficient values obtained in the current study exceeded the maximum acceptable level of 0.063 [34]. Therefore, increasing the effective population size would be essential to mitigate the risk of inbreeding.

**Table 8:** Effective population size and level of inbreeding in indigenous chickens found in different agro-ecologies of northwest Ethiopia.

Agro-ecology	Nm	Nf	Ne	ΔF
Highland	0.82	4.38	2.76	0.18
Midland	1.24	4.8	3.94	0.13
Lowland	1.44	4.18	4.28	0.12

Nm = Number of males; Nf = Number of females; Ne = Effective population size; ΔF= Inbreeding rate.

## 4 Conclusion

In conclusion, this study emphasizes that farmers prioritize egg production and growth rate when selecting chickens for income generation. Agro-ecology has a slight influence on trait preferences, management practices, and breeding objectives among households raising indigenous chickens. To achieve sustainable improvements in indigenous chicken productivity, it is crucial to consider farmers' production objectives and trait preferences within their specific production environment and agro-ecology.

## Data availability

The data relevant to this study is unrestricted and provided as supplementary material with this manuscript. The data associated with this article has not been archived in a publicly accessible repository.

## CRediT authorship contribution statement

**Bekalu Muluneh:** Writing – review & editing, Writing – original draft, Visualization, Validation, Software, Resources, Project administration, Methodology, Investigation, Funding acquisition, Formal analysis, Data curation, Conceptualization. **Mengistie Taye:** Writing – review & editing, Writing – original draft, Visualization, Validation, Supervision, Resources, Methodology, Data curation, Conceptualization. **Tadelle Dessie:** Writing – review & editing, Writing – original draft, Visualization, Validation, Supervision, Methodology, Investigation. **Dessie Salilew Wondim:** Writing – review & editing, Writing – original draft, Validation, Supervision, Methodology, Investigation. **Damitie Kebede:** Writing – review & editing, Writing – original draft, Visualization, Methodology, Investigation, Data curation. **Andualem Tenagne:** Writing – review & editing, Writing – original draft, Software, Methodology, Investigation, Formal analysis, Data curation, Conceptualization.

## Declaration of competing interest

The authors declare that they have no known competing financial interests or personal relationships that could have appeared to influence the work reported in this paper.
