# Supplementary material for: Selection criteria and husbandry practices of indigenous chicken producers in Northwest Ethiopia
Source: Heliyon. 2024 Aug 10;10(16):e36094. doi: 10.1016/j.heliyon.2024.e36094 (PMC11366869; doi:10.1016/j.heliyon.2024.e36094)
Supplement: Multimedia component 1 [file mmc1.pdf]

PA	Agro-ecology	EGG4HOME	EGG4SALE	4INCOME	4MEAT	4MANURE
1	1	1	2	4	5	
1	1	1	2	4		
1	1	2	1	3	4	8
1	1	1	2	3	4	8
1	1	4	1	2	3	8
1	1	2	1	3	4	8
1	1	2	1	3	5	8
1	1	3	1	2	5	8
1	1	1	2	3	4	8
1	1	2	1	3	4	8
1	1	3	1	2	4	7
1	1	7	1	3	4	8
1	1	1	2	4		
1	1	2	1	3	4	6
1	1	1	2	3	4	5
1	1	4	1	2	3	7
1	1	2	1	3	5	8
1	1	3	1	2	5	6
1	1	4	1	3	5	7
1	1	1	2	4		
2	1	7	1	3	8	6
2	1	1	3	2	4	6
2	1	1	3	2	5	7
2	1	1	2	3	5	6
2	1	2	1	3	4	7
2	1	2	3	1	4	7
2	1	1	2	3	4	7
2	1	1	3	4	5	6
2	1	1	3	2	4	6
2	1	1	3	2	4	6
2	1	1	3	2	5	7
2	1	1	2	3	5	6
2	1	7	1	3	8	6
2	1	2	3	1	4	7
2	1	1	2	3	4	7
2	1	1	3	4	5	6
2	1	1	2	3	5	6
2	1	7	1	3	8	6
2	1	1	2	3	5	6
2	1	7	1	3	8	6
3	1	2	1	4	7	6
3	1	2	1	4	7	6
3	1	6	2	3	7	4
3	1	2	3	4	5	6
3	1	1	2	3	4	7
3	1	5	3	4	6	2

3	1	4	3	1	5	6
3	1	3	1	2	4	6
3	1	3	2	4	5	6
3	1	1	2	3	8	7
3	1	2	3	4	5	6
3	1	4	1	3	5	6
3	1	1	2	3	5	6
3	1	7	1	3	8	6
3	1	2	1	4	7	6
3	1	2	3	4	5	6
3	1	1	2	3	4	7
3	1	5	3	4	6	2
3	1	1	2	3	4	7
3	1	5	4	2	3	6
4	1	2	1	3	7	5
4	1	2	1	4	8	6
4	1	2	1	3	5	7
4	1	5	1	2	4	6
4	1	2	1	4	8	6
4	1	4	1	2	5	7
4	1	5	1	2	4	7
4	1	3	1	2	4	8
4	1	2	1	4	8	6
4	1	2	1	3	7	5
4	1	2	1	4	8	6
4	1	3	1	2	4	6
4	1	5	4	2	3	6
4	1	4	1	3	5	6
4	1	3	1	2	5	6
4	1	2	1	3	7	5
4	1	2	1	4	8	6
4	1	2	1	3	5	7
4	1	3	1	2	5	7
4	1	3	1	2	4	7
5	1	4	1	2	6	5
5	1	2	1	3	7	8
5	1	2	1	3	4	5
5	1	2	1	4	7	5
5	1	2	1	5	4	8
5	1	2	1	4	5	6
5	1	2	1	6	4	8
5	1	2	1	5	4	8
5	1	5	3	4	6	2
5	1	2	1	4	7	6
5	1	2	1	3	6	8
5	1	4	1	2	6	5
5	1	2	1	3	7	8

5	1	2	1	3	6	8
5	1	2	1	4	7	6
5	1	2	1	3	4	8
5	1	7	1	3	8	6
5	1	2	1	4	6	7
5	1	2	1	3	6	8
5	1	3	1	2	6	7
6	1	3	1	2	7	6
6	1	2	1	3	7	6
6	1	1	2	3	6	7
6	1	5	1	4	3	6
6	1	2	1	3	4	8
6	1	2	1	3	6	8
6	1	5	2	3	4	8
6	1	3	2	1	4	8
6	1	2	1	3	7	5
6	1	2	1	3	8	6
6	1	2	1	4	8	6
6	1	2	1	4	8	5
6	1	2	1	3	7	8
6	1	2	3	4	7	6
6	1	2	1	3	7	6
6	1	1	2	4	7	8
6	1	3	1	2	7	6
6	1	2	1	3	7	6
6	1	2	1	3	7	6
6	1	4	1	2	6	5
7	2	2	1	4	7	6
7	2	5	1	2		
7	2	5	1	2	7	6
7	2	3	1	4	7	6
7	2	4	1	2	5	6
7	2	2	1	4	7	6
7	2	4	1	2	8	7
7	2	6	1	2	5	4
7	2	4	1	2		7
7	2	2	1	4	7	6
7	2	2	1	3		
7	2	2	1	3	4	7
7	2	5	1	2		
7	2	2	1	4	8	5
7	2	2	1	5	6	7
7	2	2	1	3	7	5
7	2	4	1	2	8	7
7	2	3	1	4	7	6
7	2	3	1	2	6	7
7	2	7	1	2	6	8

8	2	6	1	2	5	4
8	2	4	1	2	5	6
8	2	3	1	2	4	5
8	2	6	1	2	5	4
8	2	1	3	2	5	4
8	2	4	1	2	5	6
8	2	3	1	2	4	7
8	2	2	1	3	7	6
8	2	6	5	2	1	3
8	2	2	1	4	7	6
8	2	2	1	3	4	7
8	2	1	2	5	6	7
8	2	2	1	3	7	5
8	2	2	1	4	7	6
8	2	5	1	2		
8	2	6	5	2	1	3
8	2	2	1	4	7	6
8	2	3	1	5	4	6
8	2	2	1	4	7	6
8	2	6	5	2	1	3
9	2	4	1	2	5	6
9	2	2	1	4	7	6
9	2	5	1	2		
9	2	3	1	4	7	6
9	2	6	5	2	1	3
9	2	1	3	2	5	6
9	2	6	1	2	5	4
9	2	2	4	3	5	6
9	2	3	1	4	7	6
9	2	1	2	3	5	6
9	2	3	1	2	4	7
9	2	2	3	6	4	8
9	2	4	1	2	8	7
9	2	2	4	3	5	6
9	2	6	5	2	1	3
9	2	3	1	5	4	6
9	2	3	1	4	7	6
9	2	2	1	4	8	5
9	2	2	4	3	6	5
9	2	2	3	6	4	8
10	2	1	2	5	6	7
10	2	1	2	4	3	5
10	2	2	1	3	7	6
10	2	2	1	3	6	7
10	2	2	1	3	4	6
10	2	3	1	2	5	8
10	2	3	1	2	4	6

10	2	1	2	4	3	5
10	2	3	1	2	4	5
10	2	2	1	3	6	7
10	2	3	1	2	8	4
10	2	1	2	4	3	5
10	2	2	3	1	8	4
10	2	2	1	3	7	6
10	2	2	3	1	7	4
10	2	1	2	5	6	7
10	2	2	1	3	4	6
10	2	3	1	2	5	8
10	2	3	1	2	4	6
10	2	2	3	1	6	7
11	2	2	1	3	6	7
11	2	1	2	4	3	5
11	2	2	1	3	8	6
11	2	2	1	3	6	7
11	2	4	1	5	2	6
11	2	3	2	4	5	7
11	2	1	2	4	5	7
11	2	1	2	4	3	5
11	2	3	2	6	4	7
11	2	1	2	5	4	6
11	2	4	1	5	7	6
11	2	2	1	3	6	7
11	2	4	1	3	6	5
11	2	3	1	4	6	5
11	2	4	2	5	3	6
11	2	1	2	4	3	5
11	2	2	1	3	6	8
11	2	2	1	3	6	7
11	2	1	2	4	3	6
11	2	2	1	3	7	5
12	2	1	2	4	8	6
12	2	2	1	3	6	7
12	2	4	2	3	7	6
12	2	1	2	4	3	5
12	2	5	1	3	6	7
12	2	1	2	4	8	6
12	2	6	2	1	3	4
12	2	4	2	3	7	6
12	2	2	3	1	7	6
12	2	1	2	4	8	6
12	2	6	3	1	2	7
12	2	4	2	3	7	6
12	2	6	2	4	3	5
12	2	1	2	4	8	6

12	2	5	3	2	7	4
12	2	3	1	6	7	4
12	2	6	2	1	5	7
12	2	1	2	4	7	3
12	2	2	6	3	7	8
12	2	6	2	1	3	7
13	3	2	1	4	6	7
13	3	2	1	4	6	7
13	3	6	5	2	7	4
13	3	2	1	4	6	7
13	3	5	4	2	3	7
13	3	6	5	2	7	4
13	3	2	3	4	6	8
13	3	2	1	4	6	7
13	3	1	5	4	3	8
13	3	6	5	2	7	4
13	3	3	2	5	4	6
13	3	2	1	4	6	7
13	3	8	6	1	2	7
13	3	3	4	1	5	8
13	3	8	3	4	5	6
13	3	8	4	3	7	6
13	3	4	2	3	5	7
13	3	1	7	2	3	8
13	3	3	2	5	4	7
13	3	5	1	2	6	8
14	3	2	8	3	4	7
14	3	2	1	4	6	7
14	3	2	1	4	6	7
14	3	2	3	6	5	7
14	3	4	5	3	2	8
14	3	2	1	4	6	7
14	3	1	2	4	3	5
14	3	2	1	4	6	7
14	3	2	8	3	6	5
14	3	1	5	3	2	8
14	3	4	5	3	2	8
14	3	2	1	4	6	7
14	3	1	2	4	3	5
14	3	2	8	3	6	5
14	3	2	1	4	6	7
14	3	1	2	3	4	5
14	3	2	8	3	6	5
14	3	2	1	4	6	7
14	3	4	5	3	2	8
14	3	2	1	4	6	7
15	3	4	5	3	2	8

15	3	3	4	1	5	8
15	3	2	8	3	6	5
15	3	8	6	1	2	7
15	3	2	1	4	6	7
15	3	3	1	2	5	6
15	3	4	5	3	2	8
15	3	4	1	5	7	6
15	3	2	8	3	4	7
15	3	1	2	5	4	6
15	3	4	5	3	2	8
15	3	2	1	4	6	7
15	3	1	2	3	5	7
15	3	2	1	4	6	7
15	3	8	6	1	2	7
15	3	2	8	3	4	7
15	3	6	1	2	5	8
15	3	4	5	3	2	8
15	3	4	2	3	1	5
15	3	2	8	3	6	5
16	3	1	2	5	6	7
16	3	2	4	3	7	8
16	3	1	2	3	4	8
16	3	2	1	3	7	6
16	3	5	1	2	7	6
16	3	2	4	3	7	8
16	3	1	2	3	4	6
16	3	2	1	3	6	8
16	3	1	2	3	4	7
16	3	2	1	3	7	6
16	3	1	3	2	4	5
16	3	2	4	3	7	8
16	3	4	1	2	6	5
16	3	5	4	3	2	8
16	3	5	3	1	6	7
16	3	3	1	2	8	7
16	3	3	1	2	5	6
16	3	2	4	3	7	8
16	3	1	3	2	4	7
16	3	2	3	4	5	8
17	3	2	1	3	7	6
17	3	1	2	5	6	7
17	3	3	2	4	5	7
17	3	2	3	4	5	8
17	3	2	1	3	6	8
17	3	1	2	3	4	6
17	3	2	1	3	4	8
17	3	1	2	5	7	6

17	3	3	1	2	5	6
17	3	1	2	5	6	7
17	3	3	1	2	6	7
17	3	2	1	3	7	6
17	3	2	1	3	6	8
17	3	1	2	3	4	7
17	3	2	3	1	7	8
17	3	2	3	4	5	8
17	3	3	4	2	5	8
17	3	2	1	3	6	8
17	3	3	1	2	8	7
17	3	1	2	5	6	7
18	3	2	4	3	7	8
18	3	2	1	3	6	8
18	3	1	2	5	7	6
18	3	2	8	3	6	5
18	3	3	4	5	2	8
18	3	2	3	4	5	8
18	3	3	2	4	5	8
18	3	1	2	3	4	8
18	3	1	3	4	5	8
18	3	3	2	4	5	7
18	3	2	3	4	5	8
18	3	1	2	3	4	7
18	3	1	2	3	4	6
18	3	2	1	3	6	8
18	3	2	3	4	5	8
18	3	3	2	4	5	8
18	3	5	4	3	2	8
18	3	3	2	4	5	8
18	3	1	3	4	5	8
18	3	2	3	4	5	8

4BREEDING	4SAVING	4CEREMONY
3	6	
3	5	
5	6	7
6	7	5
5	6	7
5	6	7
4	7	6
4	6	7
5	7	6
5	6	7
5	6	8
2	5	6
3	5	
5	7	8
6	8	7
5	6	8
4	7	6
4	7	8
2	8	6
3	5	
2	4	5
5	7	8
4	6	8
4	7	8
5	6	8
5	6	8
5	6	8
2	7	8
5	8	7
5	7	8
4	6	8
4	7	8
2	4	5
5	6	8
5	6	8
2	7	8
4	7	8
2	4	5
4	7	8
2	4	5
3	5	8
3	5	8
1	5	8
1	8	7
5	8	6
1	7	8

2	7	8
5	7	8
1	8	7
4	5	6
1	8	7
2	7	8
4	7	8
2	4	5
3	5	8
1	8	7
5	8	6
1	7	8
5	8	6
1	7	8
4	6	8
3	5	7
4	6	8
3	7	8
3	5	7
3	6	8
3	6	8
5	6	7
3	5	7
4	6	8
3	5	7
5	7	8
1	7	8
2	7	8
4	7	8
4	6	8
3	5	7
4	6	8
4	6	8
5	6	8
3	7	8
6	4	5
7	6	8
3	6	8
6	3	7
3	8	7
7	3	5
6	3	7
1	7	8
3	5	8
5	4	7
3	7	8
6	4	5

5	4	7
3	5	8
7	5	6
2	4	5
3	5	8
4	5	7
4	5	8
4	5	8
4	5	8
5	4	8
7	2	8
6	5	7
4	5	7
6	1	7
6	5	7
4	6	8
4	5	7
3	5	7
3	6	7
6	4	5
5	1	8
4	5	8
3	5	6
4	5	8
4	5	8
4	5	8
3	7	8
3	5	8
4	3	
4	3	8
2	5	8
8	3	7
3	5	8
5	3	6
7	8	3
5	3	6
3	5	8
4	5	
5	6	8
4	3	
3	6	7
3	4	8
4	6	8
5	3	6
2	5	8
4	5	8
3	4	5

7	8	3
8	3	7
7	6	8
7	8	3
6	7	8
8	3	7
6	5	8
4	5	8
4	7	8
3	5	8
5	6	8
3	4	8
4	6	8
3	5	8
4	3	
4	7	8
3	5	8
2	7	8
3	5	8
4	7	8
8	3	7
3	5	8
4	3	
2	5	8
4	7	8
4	7	8
7	8	3
1	7	8
2	5	8
7	4	8
6	5	8
1	7	5
5	3	6
1	7	8
4	7	8
2	7	8
2	5	8
3	6	7
1	7	8
1	7	5
3	4	8
6	7	8
4	5	8
4	5	8
5	7	8
4	7	6
5	7	8

6	7	8
6	7	8
4	5	8
5	6	7
6	7	8
5	6	7
4	5	8
5	6	8
3	4	8
5	7	8
4	7	6
5	7	8
4	5	8
4	5	8
6	7	8
4	5	7
4	5	8
3	7	8
1	6	8
3	6	8
6	7	8
1	5	8
3	7	8
3	2	8
4	5	8
2	7	8
2	8	7
1	7	8
6	7	8
4	5	7
4	5	8
5	7	8
4	6	8
3	5	7
4	5	8
1	5	8
6	7	8
2	4	8
3	5	7
7	8	5
1	5	8
4	5	8
3	5	7
4	5	8
1	5	8
1	7	8
3	5	7

1	6	8
2	5	8
4	3	8
5	6	8
1	4	5
5	4	8
3	5	8
3	5	8
1	3	8
3	5	8
1	6	8
1	3	8
1	5	7
3	5	8
2	6	7
1	3	8
1	7	8
3	5	8
3	4	5
6	7	2
1	7	2
1	5	2
1	6	8
4	5	6
1	8	6
4	3	7
1	6	5
3	5	8
3	5	8
1	8	4
1	6	7
3	5	8
6	7	8
3	5	8
1	4	7
4	6	7
1	6	7
3	5	8
6	7	8
1	4	7
3	5	8
6	7	8
1	4	7
3	5	8
1	6	7
3	5	8
1	6	7

6	7	2
1	4	7
3	4	5
3	5	8
4	5	6
1	6	7
2	3	8
1	6	5
3	8	7
1	6	7
3	5	8
6	8	4
3	5	8
3	4	5
1	6	5
3	4	7
1	6	7
6	7	8
1	4	7
3	4	8
1	6	5
5	6	7
4	5	8
3	4	8
1	6	5
5	7	8
4	5	7
6	8	1
4	5	8
6	7	8
1	6	5
3	7	8
1	7	6
2	4	8
5	4	6
4	7	8
1	6	5
6	5	8
1	6	7
4	5	8
3	4	8
1	6	8
1	6	7
4	5	7
5	7	8
7	5	6
3	4	8

4	7	8
3	4	8
4	5	8
4	5	8
4	5	7
6	8	1
4	5	6
1	6	7
1	6	7
4	5	7
5	4	6
3	4	8
1	6	5
4	5	7
3	4	8
1	4	7
1	6	7
1	6	7
1	7	6
5	6	7
2	7	6
1	6	8
1	6	7
6	8	1
5	7	8
4	5	7
1	6	7
1	6	7
1	7	6
1	7	6
2	6	7
1	6	7
