# Supplementary material for: Selection criteria and husbandry practices of indigenous chicken producers in Northwest Ethiopia
Source: Heliyon. 2024 Aug 10;10(16):e36094. doi: 10.1016/j.heliyon.2024.e36094 (PMC11366869; doi:10.1016/j.heliyon.2024.e36094)
Supplement: Multimedia component 2 [file mmc2.pdf]

PA	Agro-ecology	MEAT	INCOME	MANURE	BREEDING	SAVING	CEREMONY
1	1	3	1	6	2	5	4
1	1	3	2	6	1	4	5
1	1	4	2	6	1	3	5
1	1	2	1	6	3	4	5
1	1	5	1	6	2	3	4
1	1	2	1	6	3	4	5
1	1	3	2	6	1	4	5
1	1	3	1	6	2	4	5
1	1	2	1	6	3	5	4
1	1	1	3	6	2	4	5
1	1	3	2	6	1	4	5
1	1	3	2	6	1	5	4
1	1	3	2	5	1	4	6
1	1	4	2	6	1	3	5
1	1	2	1	6	3	4	5
1	1	5	1	6	2	3	4
1	1	3	2	6	1	4	5
1	1	3	1	6	2	4	5
1	1	3	2	4	1	5	6
1	1	3	2	5	1	4	6
2	1	4	1	6	2	3	5
2	1	3	1	4	2	5	6
2	1	3	2	5	1	4	6
2	1	2	1	5	3	4	6
2	1	3	1	4	2	5	6
2	1	3	2	5	1	4	6
2	1	2	1	5	3	4	6
2	1	3	2	5	1	4	6
2	1	3	2	5	1	4	6
2	1	3	1	4	2	5	6
2	1	3	2	5	1	4	6
2	1	2	1	5	3	4	6
2	1	4	1	6	2	3	5
2	1	3	2	5	1	4	6
2	1	2	1	5	3	4	6
2	1	3	2	5	1	4	6
2	1	2	1	5	3	4	6
2	1	4	1	6	2	3	5
2	1	2	1	5	3	4	6
2	1	4	1	6	2	3	5
3	1	5	2	6	1	3	4
3	1	4	1	5	2	3	6
3	1	3	2	4	1	5	6
3	1	2	3	6	1	5	4
3	1	1	2	6	3	4	5
3	1	4	2	3	1	5	6

3	1	3	2	5	1	6	4
3	1	4	2	5	1	6	3
3	1	3	2	4	1	5	6
3	1	1	2	6	4	3	5
3	1	3	2	5	1	6	4
3	1	2	1	5	4	3	6
3	1	2	1	5	3	4	6
3	1	4	1	6	2	3	5
3	1	5	2	6	1	3	4
3	1	2	3	6	1	5	4
3	1	1	2	6	3	4	5
3	1	4	2	3	1	5	6
3	1	1	2	6	3	4	5
3	1	3	2	5	1	4	6
4	1	6	1	4	2	3	5
4	1	5	2	4	1	3	6
4	1	2	1	5	3	4	6
4	1	4	1	5	3	2	6
4	1	5	2	4	1	3	6
4	1	3	1	5	2	4	6
4	1	3	1	5	2	4	6
4	1	2	1	5	3	4	6
4	1	2	3	6	1	5	4
4	1	6	1	4	2	3	5
4	1	5	2	4	1	3	6
4	1	3	2	5	1	4	6
4	1	3	2	5	1	4	6
4	1	3	1	5	2	4	6
4	1	2	1	6	3	4	5
4	1	6	1	4	2	3	5
4	1	5	2	4	1	3	6
4	1	2	1	5	3	4	6
4	1	2	1	5	3	4	6
4	1	3	1	5	2	4	6
5	1	4	2	6	1	3	5
5	1	2	1	6	4	5	3
5	1	2	1	4	5	3	6
5	1	1	2	6	5	4	3
5	1	2	1	6	4	3	5
5	1	2	1	5	4	3	6
5	1	1	3	4	6	2	5
5	1	2	1	6	4	3	5
5	1	4	2	3	1	5	6
5	1	5	2	6	1	3	4
5	1	1	2	6	3	4	5
5	1	4	2	6	1	3	5
5	1	2	1	6	4	5	3

5	1	1	3	5	2	4	6
5	1	5	2	6	1	3	4
5	1	2	1	6	5	3	4
5	1	4	1	6	2	3	5
5	1	2	1	6	5	3	4
5	1	1	3	6	4	2	5
5	1	3	1	6	5	2	4
6	1	4	2	6	1	5	3
6	1	5	2	6	1	4	3
6	1	1	2	4	3	5	6
6	1	1	2	5	3	4	6
6	1	1	2	3	4	5	6
6	1	1	4	6	2	3	5
6	1	5	4	6	1	3	2
6	1	3	1	6	4	2	5
6	1	6	1	4	2	3	5
6	1	2	1	6	4	3	5
6	1	5	2	4	1	3	6
6	1	3	2	4	5	1	6
6	1	2	1	6	4	5	3
6	1	2	3	6	4	1	5
6	1	5	2	6	1	4	3
6	1	3	4	6	1	2	5
6	1	4	2	6	1	5	3
6	1	5	2	6	1	4	3
6	1	3	2	5	4	1	6
6	1	4	2	6	1	3	5
7	2	3	4	6	1	5	2
7	2	4	1		5	2	3
7	2	3	1	6	4	2	5
7	2	5	2	6	1	4	3
7	2	3	1	5	4	2	6
7	2	3	4	6	1	5	2
7	2	4	1	6	2	5	3
7	2	5	2	4	1	3	6
7	2	3	1	6	4	2	5
7	2	3	4	6	1	5	2
7	2	4	1	5	6	2	3
7	2	2	1	3	4	5	6
7	2	4	1		5	2	3
7	2	5	2	3	1	4	6
7	2	5	2	6	3	1	4
7	2	2	1	6	3	4	5
7	2	2	1	6	3	4	5
7	2	5	2	6	1	4	3
7	2	4	1	6	2	3	5
7	2	3	1	6	4	2	5

8	2	5	2	4	1	3	6
8	2	3	1	5	4	2	6
8	2	2	3	4	5	1	6
8	2	5	2	4	1	3	6
8	2	5	2	3	4	6	1
8	2	3	1	5	4	2	6
8	2	2	1	5	4	3	6
8	2	3	2	5	1	4	6
8	2	5	2	4	1	3	6
8	2	3	4	6	1	5	2
8	2	2	1	3	4	5	6
8	2	3	2	6	1	5	4
8	2	2	1	6	3	4	5
8	2	3	4	6	1	5	2
8	2	4	1		5	2	3
8	2	5	2	4	1	3	6
8	2	3	4	6	1	5	2
8	2	2	1	6	3	5	4
8	2	3	4	6	1	5	2
8	2	5	2	4	1	3	6
9	2	3	1	5	4	2	6
9	2	3	4	6	1	5	2
9	2	4	1		5	2	3
9	2	5	2	6	1	4	3
9	2	5	2	4	1	3	6
9	2	1	3	4	2	5	6
9	2	5	2	4	1	3	6
9	2	1	3	4	2	5	6
9	2	5	2	6	1	4	3
9	2	2	1	5	3	6	4
9	2	2	1	5	4	3	6
9	2	1	2	5	3	4	6
9	2	4	1	6	2	5	3
9	2	3	2	6	1	4	5
9	2	5	2	4	1	3	6
9	2	2	1	6	3	5	4
9	2	5	2	6	1	4	3
9	2	5	2	3	1	4	6
9	2	1	3	4	2	5	6
9	2	4	2	6	1	5	3
10	2	3	2	6	1	5	4
10	2	2	1	3	4	5	6
10	2	3	2	5	1	4	6
10	2	3	2	6	1	4	5
10	2	2	1	3	4	5	6
10	2	3	2	6	1	5	4
10	2	2	1	4	3	5	6

10	2	2	1	3	4	5	6
10	2	4	1	3	2	5	6
10	2	3	2	6	1	4	5
10	2	4	3	6	1	2	5
10	2	2	1	3	4	5	6
10	2	5	1	3	2	4	6
10	2	3	2	5	1	4	6
10	2	5	1	2	4	3	6
10	2	3	2	6	1	5	4
10	2	2	1	4	3	5	6
10	2	3	2	6	1	5	4
10	2	2	1	4	3	5	6
10	2	5	1	6	2	3	4
11	2	3	2	6	1	4	5
11	2	2	1	3	4	5	6
11	2	3	1	6	2	4	5
11	2	3	2	6	1	4	5
11	2	3	2	4	1	5	6
11	2	3	2	5	1	4	6
11	2	3	2	6	1	4	5
11	2	2	1	3	4	5	6
11	2	3	2	5	1	4	6
11	2	1	2	3	5	4	6
11	2	3	2	5	1	4	6
11	2	3	2	6	1	4	5
11	2	3	2	4	1	5	6
11	2	3	2	6	1	4	5
11	2	3	2	4	1	5	6
11	2	2	1	3	4	5	6
11	2	4	2	3	1	5	6
11	2	3	2	6	1	4	5
11	2	2	1	3	4	6	5
11	2	3	2	4	1	5	6
12	2	4	1	6	2	5	3
12	2	3	2	6	1	4	5
12	2	4	2	6	1	3	5
12	2	2	1	3	4	5	6
12	2	2	1	3	4	5	6
12	2	4	1	6	2	5	3
12	2	2	4	5	1	6	3
12	2	4	2	6	1	3	5
12	2	4	2	5	3	1	7
12	2	4	1	6	2	5	3
12	2	1	2	6	3	4	5
12	2	4	2	6	1	3	5
12	2	4	1	5	2	3	6
12	2	4	1	6	2	5	3

12	2	3	2	5	1	4	6
12	2	4	2	5	1	3	6
12	2	4	1	6	3	2	5
12	2	4	1	5	1	7	6
12	2	2	4	5	1	3	6
12	2	4	2	5	1	3	6
13	3	4	2	6	1	3	5
13	3	4	2	6	1	3	5
13	3	5	1	4	2	3	6
13	3	4	2	6	1	3	5
13	3	2	3	6	1	5	4
13	3	5	1	4	2	3	6
13	3	5	3	6	4	1	2
13	3	4	2	6	1	3	5
13	3	2	3	6	5	4	1
13	3	5	1	4	2	3	6
13	3	2	3	6	1	4	5
13	3	4	2	6	1	3	5
13	3	5	3	6	1	2	4
13	3	1	2	6	3	4	5
13	3	1	3	5	2	6	4
13	3	4	2	6	3	5	1
13	3	4	2	5	1	6	3
13	3	1	3	6	4	5	2
13	3	4	1	6	5	2	3
13	3	3	2	6	1	4	5
14	3	3	1	5	2	4	6
14	3	4	2	6	1	3	5
14	3	4	2	6	1	3	5
14	3	3	1	6	4	5	2
14	3	3	4	6	1	5	2
14	3	4	2	6	1	3	5
14	3	1	2	5	3	4	6
14	3	4	2	6	1	3	5
14	3	1	2	6	3	4	5
14	3	1	2	6	3	4	5
14	3	3	4	6	1	5	2
14	3	4	2	6	1	3	5
14	3	1	2	4	3	5	6
14	3	1	2	6	3	4	5
14	3	4	2	6	1	3	5
14	3	1	2	3	4	5	6
14	3	1	2	6	3	4	5
14	3	4	2	6	1	3	5
14	3	3	4	6	1	5	2
14	3	4	2	6	1	3	5
15	3	3	4	6	1	5	2

15	3	1	2	6	3	4	5
15	3	1	2	6	3	4	5
15	3	5	3	6	1	2	4
15	3	4	2	6	1	3	5
15	3	2	1	5	4	6	3
15	3	3	4	6	1	5	2
15	3	4	2	5	3	1	6
15	3	3	1	5	2	4	6
15	3	3	2	5	1	6	4
15	3	3	4	6	1	5	2
15	3	4	2	6	1	3	5
15	3	2	1	6	3	4	6
15	3	4	2	6	1	3	5
15	3	5	3	6	1	2	4
15	3	3	1	5	2	4	6
15	3	2	4	5	1	3	6
15	3	3	4	6	1	5	2
15	3	1	3	4	2	5	6
15	3	1	2	6	3	4	5
16	3	1	2	6	3	4	5
16	3	1	3	5	2	4	6
16	3	1	2	5	3	4	6
16	3	2	1	6	4	3	5
16	3	3	1	6	2	4	5
16	3	1	3	5	2	4	6
16	3	1	2	4	3	5	6
16	3	2	1	7	3	4	8
16	3	2	1	3	5	4	6
16	3	2	1	6	4	3	5
16	3	1	2	4	3	5	6
16	3	1	3	5	2	4	6
16	3	1	2	4	3	5	6
16	3	3	2	6	1	4	5
16	3	4	2	5	1	3	6
16	3	3	1	6	4	2	5
16	3	1	2	5	3	4	6
16	3	1	3	5	2	4	6
16	3	1	2	4	3	5	6
16	3	2	1	6	3	5	4
17	3	2	1	6	4	3	5
17	3	1	2	6	3	4	5
17	3	3	2	5	1	4	6
17	3	2	1	6	3	5	4
17	3	2	1	6	3	4	5
17	3	1	2	5	3	4	6
17	3	2	1	6	3	5	4
17	3	1	4	3	2	5	6

17	3	2	1	6	4	5	3
17	3	1	2	6	3	4	5
17	3	4	2	6	3	5	1
17	3	2	1	6	4	3	5
17	3	2	1	7	3	4	8
17	3	2	1	3	5	4	6
17	3	3	2	5	1	4	6
17	3	2	1	6	3	5	4
17	3	3	2	6	1	4	5
17	3	2	1	7	3	4	8
17	3	3	1	6	4	2	5
17	3	1	2	6	3	4	5
18	3	1	3	5	2	4	6
18	3	2	1	7	3	4	8
18	3	1	4	3	2	5	6
18	3	1	2	6	3	4	5
18	3	2	4	6	3	5	1
18	3	2	1	6	3	5	4
18	3	1	2	6	3	5	4
18	3	1	2	5	3	4	6
18	3	1	2	6	3	5	4
18	3	3	2	5	1	4	6
18	3	1	2	6	3	5	4
18	3	2	1	3	5	4	6
18	3	1	2	5	3	4	6
18	3	2	1	7	3	4	8
18	3	2	1	6	3	5	4
18	3	1	2	6	3	5	4
18	3	3	2	6	1	4	5
18	3	3	1	6	2	5	4
18	3	1	2	6	3	4	5
18	3	2	1	6	3	5	4
