# Supplementary material for: Selection criteria and husbandry practices of indigenous chicken producers in Northwest Ethiopia
Source: Heliyon. 2024 Aug 10;10(16):e36094. doi: 10.1016/j.heliyon.2024.e36094 (PMC11366869; doi:10.1016/j.heliyon.2024.e36094)
Supplement: Multimedia component 3 [file mmc3.pdf]

PA	Agro-ecology	WHITE	RED	BLACK	GEBISMA	TETERIMA
1	1	4	1	5	2	3
1	1	4	1	5	2	3
1	1	3	1	4	2	5
1	1	4	1	3	2	5
1	1	4	1	3	2	5
1	1	3	1	4	2	5
1	1	3	1	4	2	5
1	1	2	1	3	4	5
1	1	4	1	3	2	5
1	1	2	1	3	4	5
1	1	4	1	3	2	5
1	1	3	1	4	2	5
1	1	4	1	5	2	3
1	1	2	1	5	3	4
1	1	5	1	3	2	4
1	1	4	1	3	2	5
1	1	5	1	4	2	3
1	1	2	1	3	4	5
1	1	3	1	5	2	4
1	1	5	1	4	2	3
2	1	2	1	5	3	4
2	1	2	1	4	3	5
2	1	2	1	4	3	5
2	1	2	1	4	5	3
2	1	2	1	4	3	5
2	1	2	1	5	3	4
2	1	3	1	4	2	5
2	1	2	1	5	3	4
2	1	2	1	3	5	4
2	1	2	1	4	3	5
2	1	2	1	4	3	5
2	1	2	1	4	5	3
2	1	2	1	5	3	4
2	1	2	1	5	3	4
2	1	3	1	4	2	5
2	1	2	1	5	3	4
2	1	2	1	4	5	3
2	1	2	1	5	3	4
2	1	2	1	4	5	3
2	1	2	1	5	3	4
3	1	4	1	5	2	3
3	1	2	1	5	3	4
3	1	3	2	5	1	4
3	1	2	3	4	1	5
3	1	3	1	4	2	5
3	1	3	2	4	1	5

3	1	4	1	3	2	5
3	1	4	2	3	1	5
3	1	4	1	2	3	5
3	1	3	2	4	1	5
3	1	3	1	4	2	5
3	1	2	1	4	3	5
3	1	2	1	4	5	3
3	1	2	1	5	3	4
3	1	4	1	5	2	3
3	1	2	3	4	1	5
3	1	3	1	4	2	5
3	1	3	2	4	1	5
3	1	3	1	4	2	5
3	1	4	1	3	2	5
4	1	2	1	5	3	4
4	1	3	2	5	1	4
4	1	2	1	5	3	4
4	1	2	1	4	3	5
4	1	2	1	4	3	5
4	1	2	1	3	4	5
4	1	2	1	4	3	5
4	1	2	1	4	3	5
4	1	3	1	5	2	4
4	1	2	1	5	3	4
4	1	3	2	5	1	4
4	1	2	1	5	4	3
4	1	4	1	3	2	5
4	1	2	1	4	3	5
4	1	2	1	4	3	5
4	1	2	1	5	3	4
4	1	3	2	5	1	4
4	1	2	1	5	3	4
4	1	2	1	3	4	5
4	1	2	1	4	3	5
5	1	1	3	5	2	4
5	1	1	2	5	3	4
5	1	5	1	4	2	3
5	1	5	1	4	2	3
5	1	3	4	1	2	5
5	1	2	1	5	3	4
5	1	2	1	3	4	5
5	1	5	1	4	2	3
5	1	3	2	4	1	5
5	1	4	1	5	2	3
5	1	1	2	5	3	4
5	1	1	3	5	2	4
5	1	1	2	5	3	4

5	1	5	1	2	3	4
5	1	4	1	5	2	3
5	1	1	2	5	3	4
5	1	2	1	5	3	4
5	1	5	2	1	3	4
5	1	5	1	2	3	4
5	1	2	1	5	3	4
6	1	2	1	5	3	4
6	1	3	1	5	2	4
6	1	5	1	2	3	4
6	1	4	1	2	3	5
6	1	5	1	2	3	4
6	1	5	3	4	1	2
6	1	5	1	4	2	3
6	1	5	2	1	3	4
6	1	2	1	5	3	4
6	1	5	1	2	3	4
6	1	3	2	5	1	4
6	1	5	1	2	4	3
6	1	1	2	5	3	4
6	1	5	1	2	4	3
6	1	3	1	5	2	4
6	1	4	1	5	2	3
6	1	2	1	5	3	4
6	1	3	1	5	2	4
6	1	5	1	2	3	4
6	1	1	3	5	2	4
7	2	2	1	5	3	4
7	2	2	1	5	3	4
7	2	2	1	5	3	4
7	2	4	1	5	2	3
7	2	1	2	5	3	4
7	2	2	1	5	3	4
7	2	2	1	4	3	5
7	2	2	1	3	5	4
7	2	2	1	5	3	4
7	2	2	1	5	3	4
7	2	2	1	5	3	4
7	2	2	1	3	5	4
7	2	2	1	5	3	4
7	2	2	1	5	3	4
7	2	2	1	5	3	4
7	2	2	3	4	5	1
7	2	2	1	5	4	3
7	2	4	1	5	2	3
7	2	2	1	5	4	3
7	2	2	1	5	3	4

8	2	2	1	3	5	4
8	2	1	2	5	3	4
8	2	2	3	1	4	5
8	2	2	1	3	5	4
8	2	2	5	3	4	1
8	2	1	2	5	3	4
8	2	1	4	3	2	5
8	2	1	2	5	3	4
8	2	2	3	1	4	5
8	2	2	1	5	3	4
8	2	2	1	3	5	4
8	2	3	1	2	4	5
8	2	2	3	4	5	1
8	2	2	1	5	3	4
8	2	2	1	5	3	4
8	2	2	3	1	4	5
8	2	2	1	5	3	4
8	2	2	1	3	5	4
8	2	2	1	5	3	4
8	2	2	3	1	4	5
9	2	1	2	5	3	4
9	2	2	1	5	3	4
9	2	2	1	5	3	4
9	2	4	1	5	2	3
9	2	2	3	1	4	5
9	2	2	1	5	3	4
9	2	2	1	3	5	4
9	2	2	1	5	3	4
9	2	4	1	5	2	3
9	2	2	1	5	3	4
9	2	1	4	3	2	5
9	2	2	1	5	3	4
9	2	2	1	4	3	5
9	2	2	1	5	3	4
9	2	2	3	1	4	5
9	2	2	1	3	5	4
9	2	4	1	5	2	3
9	2	2	1	5	3	4
9	2	2	1	5	3	4
9	2	2	1	5	3	4
10	2	3	1	2	4	5
10	2	1	2	3	4	5
10	2	1	2	5	3	4
10	2	1	2	5	3	4
10	2	2	1	4	3	5
10	2	1	2	4	3	5
10	2	2	1	4	3	5

10	2	1	2	3	4	5
10	2	2	1	3	4	5
10	2	1	2	5	3	4
10	2	2	1	4	3	5
10	2	1	2	3	4	5
10	2	2	3	4	1	5
10	2	1	2	5	3	4
10	2	1	3	2	4	5
10	2	3	1	2	4	5
10	2	2	1	4	3	5
10	2	1	2	4	3	5
10	2	1	3	4	2	5
10	2	1	2	4	3	5
11	2	1	2	5	3	4
11	2	1	2	3	4	5
11	2	1	2	5	3	4
11	2	1	2	5	3	4
11	2	2	1	5	3	4
11	2	3	1	5	2	4
11	2	1	4	2	3	5
11	2	1	2	3	4	5
11	2	1	2	3	4	5
11	2	1	2	4	3	5
11	2	2	1	5	3	4
11	2	1	2	5	3	4
11	2	1	3	5	2	4
11	2	2	1	4	3	5
11	2	1	2	5	3	4
11	2	1	2	3	4	5
11	2	2	1	4	3	5
11	2	1	2	5	3	4
11	2	2	3	1	4	5
11	2	2	1	3	4	5
12	2	1	2	5	3	4
12	2	1	2	5	3	4
12	2	1	2	5	3	4
12	2	1	2	3	4	5
12	2	2	4	1	5	3
12	2	1	2	5	3	4
12	2	2	1	4	5	3
12	2	1	2	5	3	4
12	2	2	1	4	5	3
12	2	1	2	5	3	4
12	2	2	4	1	5	3
12	2	1	2	5	3	4
12	2	1	2	3	4	5
12	2	1	2	5	3	4

12	2	2	1	4	5	3
12	2	2	4	1	5	3
12	2	2	1	4	5	3
12	2	2	1	4	3	5
12	2	2	1	4	5	3
12	2	2	1	4	5	3
13	3	5	2	4	1	3
13	3	5	2	4	1	3
13	3	4	2	5	3	1
13	3	5	2	4	1	3
13	3	1	2	4	3	5
13	3	4	2	5	3	1
13	3	4	2	5	3	1
13	3	5	2	4	1	3
13	3	4	3	5	2	1
13	3	4	2	5	3	1
13	3	3	2	1	4	5
13	3	5	2	4	1	3
13	3	1	4	5	2	3
13	3	5	1	4	3	2
13	3	4	2	5	3	1
13	3	5	1	4	3	2
13	3	4	1	5	3	2
13	3	3	1	5	2	4
13	3	4	1	5	3	2
13	3	4	2	5	1	3
14	3	4	1	5	2	3
14	3	5	2	4	1	3
14	3	5	2	4	1	3
14	3	5	1	4	2	3
14	3	2	1	5	3	4
14	3	5	2	4	1	3
14	3	2	1	5	4	3
14	3	5	2	4	1	3
14	3	4	1	5	3	2
14	3	2	1	5	3	4
14	3	2	1	5	3	4
14	3	5	2	4	1	3
14	3	2	1	5	3	4
14	3	4	1	5	3	2
14	3	5	2	4	1	3
14	3	5	1	4	2	3
14	3	4	1	5	3	2
14	3	5	2	4	1	3
14	3	2	1	5	3	4
14	3	5	2	4	1	3
15	3	2	1	5	3	4

15	3	5	1	4	3	2
15	3	4	1	5	3	2
15	3	1	4	5	2	3
15	3	5	2	4	1	3
15	3	5	4	1	3	2
15	3	2	1	5	3	4
15	3	2	1	5	4	3
15	3	4	1	5	2	3
15	3	1	2	3	4	5
15	3	2	1	5	3	4
15	3	5	2	4	1	3
15	3	1	2	5	4	3
15	3	5	2	4	1	3
15	3	1	4	5	2	3
15	3	4	1	5	2	3
15	3	1	2	5	4	3
15	3	2	1	5	3	4
15	3	1	2	3	5	4
15	3	4	1	5	3	2
16	3	4	1	5	2	3
16	3	5	2	4	1	3
16	3	5	3	2	1	4
16	3	5	1	4	2	3
16	3	3	4	5	2	1
16	3	5	2	4	1	3
16	3	3	4	2	1	5
16	3	4	1	5	3	2
16	3	3	4	5	2	1
16	3	5	1	4	2	3
16	3	2	4	3	5	1
16	3	5	2	4	1	3
16	3	1	3	4	5	2
16	3	5	1	4	3	2
16	3	3	1	4	5	2
16	3	4	2	5	3	1
16	3	2	4	5	3	1
16	3	5	2	4	1	3
16	3	4	5	2	1	3
16	3	5	1	4	2	3
17	3	5	1	4	2	3
17	3	4	1	5	2	3
17	3	4	1	5	2	3
17	3	5	1	4	2	3
17	3	4	1	5	4	2
17	3	2	1	5	3	4
17	3	4	1	5	2	3
17	3	5	1	4	2	3

17	3	4	1	5	3	2
17	3	4	1	5	2	3
17	3	4	1	5	3	2
17	3	5	1	4	2	3
17	3	4	1	5	3	2
17	3	3	4	5	2	1
17	3	4	1	5	3	2
17	3	5	1	4	2	3
17	3	4	1	5	3	2
17	3	4	1	5	3	2
17	3	4	2	5	3	1
17	3	4	1	5	2	3
18	3	5	2	4	1	3
18	3	4	1	5	3	2
18	3	5	1	4	2	3
18	3	4	1	5	3	2
18	3	4	1	5	2	3
18	3	5	1	4	2	3
18	3	5	1	4	2	3
18	3	5	3	2	1	4
18	3	5	1	4	2	3
18	3	4	1	5	2	3
18	3	5	1	4	2	3
18	3	3	4	5	2	1
18	3	2	1	5	3	4
18	3	4	1	5	3	2
18	3	5	1	4	2	3
18	3	5	1	4	2	3
18	3	5	1	4	3	2
18	3	5	1	4	2	3
18	3	5	1	4	2	3
18	3	5	1	4	2	3
