# Supplementary material for: Selection criteria and husbandry practices of indigenous chicken producers in Northwest Ethiopia
Source: Heliyon. 2024 Aug 10;10(16):e36094. doi: 10.1016/j.heliyon.2024.e36094 (PMC11366869; doi:10.1016/j.heliyon.2024.e36094)
Supplement: Multimedia component 4 [file mmc4.pdf]

PA	Agro-ecology	EGGNO	BROODY	EGG FERTILITY	PLUMAGE COLOUR	APPEARANCE
1	1	2	1	8	7	5
1	1	1	9	7	2	6
1	1	3	6	4	8	2
1	1	1	10	3	8	2
1	1	1	6	2	7	3
1	1	1	5	4	10	2
1	1	2	4	3	5	1
1	1	1	2	5	6	3
1	1	5	6	4	3	1
1	1	1	7	4	6	2
1	1	2	4	3	9	1
1	1	3	2	4	5	1
1	1	1	9	7	2	6
1	1	3	6	4	8	2
1	1	1	10	3	8	2
1	1	1	6	2	7	3
1	1	2	4	3	5	1
1	1	1	2	5	6	3
1	1	3	2	4	5	1
1	1	1	9	7	2	6
2	1	2	5	6	1	3
2	1	2	3	1	4	6
2	1	1	5	2	6	7
2	1	1	4	2	5	7
2	1	7	8	2	5	4
2	1	1	5	6	7	8
2	1	2	3	1	4	5
2	1	1	5	2	6	7
2	1	1	3	2	6	5
2	1	2	3	1	4	6
2	1	1	5	2	6	7
2	1	1	4	2	5	7
2	1	2	5	6	1	3
2	1	1	5	6	7	8
2	1	2	3	1	4	5
2	1	1	5	2	6	7
2	1	1	4	2	5	7
2	1	2	5	6	1	3
2	1	1	4	2	5	7
2	1	2	5	6	1	3
3	1	3	7	8	4	1
3	1	2	9	7	5	3
3	1	5	6	4	3	1
3	1	5	4	6	1	3
3	1	1	6	5	2	9
3	1	3	4	5	2	6

3	1	3	4	5	2	9
3	1	4	7	5	1	3
3	1	3	5	4	2	6
3	1	1	4	2	3	7
3	1	1	4	5	2	6
3	1	5	6	7	8	9
3	1	1	4	2	5	7
3	1	2	5	6	1	3
3	1	3	7	8	4	1
3	1	5	4	6	1	3
3	1	1	6	5	2	9
3	1	3	4	5	2	6
3	1	1	6	5	2	9
3	1	2	3	5	4	6
4	1	1	10	4	5	2
4	1	1	7	8	3	5
4	1	3	10	7	2	1
4	1	3	10	6	5	1
4	1	1	9	7	4	2
4	1	2	6	5	7	1
4	1	2	9	7	6	1
4	1	2	9	6	7	1
4	1	1	2	6	7	5
4	1	1	10	4	5	2
4	1	1	7	8	3	5
4	1	4	9	7	10	1
4	1	2	3	5	4	6
4	1	2	9	6	3	1
4	1	2	8	9	3	1
4	1	1	10	4	5	2
4	1	1	7	8	3	5
4	1	3	10	7	2	1
4	1	2	10	7	5	1
4	1	4	10	9	2	1
5	1	1	6	7	3	8
5	1	3	4	6	5	1
5	1	3	4	10	5	1
5	1	1	3	10	4	2
5	1	4	5	1	6	2
5	1	2	3	9	8	1
5	1	2	3	10	9	1
5	1	2	4	10	9	1
5	1	3	4	5	2	6
5	1	3	7	8	4	1
5	1	1	5	2	4	3
5	1	1	6	7	3	8
5	1	3	4	6	5	1

5	1	1	2	6	7	3
5	1	3	7	8	4	1
5	1	1	4	10	9	2
5	1	2	5	6	1	3
5	1	1	6	8	4	3
5	1	1	2	5	4	3
5	1	4	5	10	1	3
6	1	1	7	9	2	6
6	1	1	8	7	5	6
6	1	8	9	1	2	3
6	1	1	8	2	7	5
6	1	5	9	1	8	2
6	1	8	10	7	3	4
6	1	4	10	3	2	1
6	1	3	7	1	6	5
6	1	1	10	4	5	2
6	1	1	10	4	3	2
6	1	1	7	8	3	5
6	1	3	10	1	2	4
6	1	3	4	6	5	1
6	1	1	7	2	3	8
6	1	1	8	7	5	6
6	1	5	10	4	1	2
6	1	1	7	9	2	6
6	1	1	8	7	5	6
6	1	1	8	6	7	5
6	1	1	6	7	3	8
7	2	1	3	4	2	6
7	2	4	5	6	8	7
7	2	3	2	1	8	9
7	2	1	7	8	3	6
7	2	3	4	5	9	6
7	2	1	3	4	2	6
7	2	3	5	4	10	2
7	2	5	1	3	2	6
7	2	1	8	5	7	6
7	2	1	3	4	2	6
7	2	1	3	7	10	6
7	2	5	2	1	4	6
7	2	4	5	6	8	7
7	2	4	6	1	5	2
7	2	1	2	8	6	3
7	2	5	4	2	1	3
7	2	1	5	7	6	2
7	2	1	7	8	3	6
7	2	1	10	9	6	5
7	2	1	10	6	4	2

8	2	5	1	3	2	6
8	2	3	4	5	9	6
8	2	5	6	2	1	7
8	2	5	1	3	2	6
8	2	8	1	3	2	5
8	2	3	4	5	9	6
8	2	5	1	2	3	6
8	2	1	8	7	3	5
8	2	5	6	1	2	3
8	2	1	3	4	2	6
8	2	5	2	1	4	6
8	2	1	5	8	7	3
8	2	5	4	2	1	3
8	2	1	3	4	2	6
8	2	4	5	6	8	7
8	2	5	6	1	2	3
8	2	1	3	4	2	6
8	2	1	5	8	7	2
8	2	1	3	4	2	6
8	2	5	6	1	2	3
9	2	3	4	5	9	6
9	2	1	3	4	2	6
9	2	4	5	6	8	7
9	2	1	7	8	3	6
9	2	5	6	1	2	3
9	2	1	4	2	5	7
9	2	5	1	3	2	6
9	2	1	4	2	5	7
9	2	1	7	8	3	6
9	2	1	8	6	5	4
9	2	5	1	2	3	6
9	2	4	10	5	2	1
9	2	3	5	4	10	2
9	2	1	8	6	5	4
9	2	5	6	1	2	3
9	2	1	5	8	7	2
9	2	1	7	8	3	6
9	2	4	6	1	5	2
9	2	1	4	2	5	7
9	2	4	10	5	2	1
10	2	1	5	8	7	3
10	2	1	3	2	5	4
10	2	1	8	7	3	5
10	2	1	7	5	6	2
10	2	1	2	3	4	5
10	2	1	5	6	3	4
10	2	1	2	3	4	5

10	2	1	3	2	5	4
10	2	1	4	5	6	2
10	2	1	7	5	6	2
10	2	4	5	6	2	3
10	2	1	3	2	5	4
10	2	1	4	5	3	2
10	2	1	8	7	3	5
10	2	1	5	6	3	4
10	2	1	5	8	7	3
10	2	1	2	3	4	5
10	2	1	5	6	3	4
10	2	1	2	3	4	5
10	2	1	5	6	3	4
11	2	1	7	5	6	2
11	2	1	3	2	5	4
11	2	1	7	6	4	5
11	2	1	7	5	6	2
11	2	1	7	9	2	3
11	2	2	6	1	8	4
11	2	1	3	9	6	4
11	2	1	3	2	5	4
11	2	1	3	2	8	5
11	2	1	5	6	3	4
11	2	1	5	10	6	2
11	2	1	7	5	6	2
11	2	1	5	10	2	6
11	2	1	7	2	4	8
11	2	2	4	8	6	1
11	2	1	3	2	5	4
11	2	2	3	1	5	4
11	2	1	7	5	6	2
11	2	1	2	3	6	5
11	2	1	3	2	8	6
12	2	1	2	7	6	5
12	2	1	7	5	6	2
12	2	1	2	8	5	6
12	2	1	3	2	5	4
12	2	3	8	9	6	1
12	2	1	2	7	6	5
12	2	3	1	2	6	7
12	2	1	2	8	5	6
12	2	3	6	8	10	1
12	2	1	2	7	6	5
12	2	2	3	1	4	6
12	2	1	2	8	5	6
12	2	3	2	1	4	8
12	2	1	2	7	6	5

12	2	1	3	4	6	8
12	2	3	8	9	6	1
12	2	3	2	4	1	5
12	2	2	1	3	6	8
12	2	3	6	8	1	9
12	2	3	4	1	2	6
13	3	3	2	6	7	1
13	3	3	2	6	7	1
13	3	4	6	8	1	2
13	3	3	2	6	7	1
13	3	2	3	10	4	1
13	3	4	6	8	1	2
13	3	9	3	2	1	5
13	3	3	2	6	7	1
13	3	4	9	8	1	2
13	3	4	6	8	1	2
13	3	5	6	1	3	2
13	3	3	2	6	7	1
13	3	10	9	1	2	8
13	3	6	8	7	2	1
13	3	7	8	2	1	3
13	3	6	1	7	4	3
13	3	5	1	6	2	10
13	3	2	5	4	1	6
13	3	5	4	6	2	1
13	3	6	5	10	7	4
14	3	1	2	10	3	4
14	3	3	2	6	7	1
14	3	3	2	6	7	1
14	3	2	1	4	3	6
14	3	2	6	10	5	3
14	3	3	2	6	7	1
14	3	1	2	10	3	4
14	3	3	2	6	7	1
14	3	2	10	7	3	1
14	3	1	2	8	6	3
14	3	2	6	10	5	3
14	3	3	2	6	7	1
14	3	1	2	9	3	4
14	3	2	10	7	3	1
14	3	3	2	6	7	1
14	3	1	2	6	3	4
14	3	2	10	7	3	1
14	3	3	2	6	7	1
14	3	2	6	10	5	3
14	3	3	2	6	7	1
15	3	2	6	10	5	3

15	3	6	8	7	2	1
15	3	2	10	7	3	1
15	3	10	9	1	2	8
15	3	3	2	6	7	1
15	3	4	7	5	6	9
15	3	2	6	10	5	3
15	3	4	10	5	9	8
15	3	1	2	10	3	4
15	3	2	10	1	6	7
15	3	2	6	10	5	3
15	3	3	2	6	7	1
15	3	1	5	10	4	8
15	3	3	2	6	7	1
15	3	10	9	1	2	8
15	3	1	2	10	3	4
15	3	1	4	8	5	6
15	3	2	6	10	5	3
15	3	1	5	3	7	6
15	3	2	10	7	3	1
16	3	5	2	6	3	4
16	3	2	4	5	1	9
16	3	1	3	10	2	5
16	3	1	5	6	2	4
16	3	3	1	2	4	5
16	3	2	4	5	1	9
16	3	3	1	2	4	5
16	3	3	7	8	1	2
16	3	2	3	1	4	5
16	3	1	5	6	2	4
16	3	3	1	2	6	5
16	3	2	4	5	1	9
16	3	1	3	2	5	4
16	3	5	6	7	1	2
16	3	1	5	6	4	3
16	3	3	5	6	4	1
16	3	1	2	3	4	7
16	3	2	4	5	1	9
16	3	3	1	2	4	6
16	3	5	1	2	3	4
17	3	1	5	6	2	4
17	3	5	2	6	3	4
17	3	4	9	10	1	3
17	3	5	4	3	1	2
17	3	4	5	10	3	1
17	3	3	1	2	5	4
17	3	5	6	10	3	1
17	3	1	3	2	8	4

17	3	2	7	8	3	1
17	3	5	2	6	3	4
17	3	1	6	2	5	3
17	3	1	5	6	2	4
17	3	3	7	8	1	2
17	3	2	3	1	4	5
17	3	3	8	10	2	1
17	3	5	4	3	1	2
17	3	4	9	10	1	3
17	3	3	7	8	1	2
17	3	3	5	6	4	1
17	3	5	2	6	3	4
18	3	2	4	5	1	9
18	3	3	7	8	1	2
18	3	1	3	2	8	4
18	3	2	10	7	3	1
18	3	6	5	4	1	3
18	3	5	4	3	1	2
18	3	4	5	6	1	2
18	3	1	3	10	2	5
18	3	5	4	6	1	2
18	3	4	9	10	1	3
18	3	6	1	5	2	4
18	3	2	3	1	4	5
18	3	3	1	2	5	4
18	3	3	7	8	1	2
18	3	5	1	2	3	4
18	3	5	4	6	1	3
18	3	5	6	7	1	2
18	3	5	6	7	1	3
18	3	6	4	5	1	2
18	3	5	4	3	1	2

COMB TYPE	SCAVENGING ABILITY	Dx RESISTANCE	LONGEVITY	MOTHERING ABILITY
6	9	10	3	4
3	8	10	5	4
1	10	5	9	7
4	7	5	9	6
4	10	5	9	8
3	9	6	8	7
6	9	7	8	10
4	9	8	7	10
2	9	7	8	10
5	10	3	8	9
5	10	6	8	7
6	8	7	9	10
3	8	10	5	4
1	10	5	9	7
4	7	5	9	6
4	10	5	9	8
6	9	7	8	10
4	9	8	7	10
6	8	7	9	10
3	8	10	5	4
4	10	9	8	7
5	8	7	10	9
4	8	3	10	9
6	8	3	10	9
3	6	1	10	9
2	4	3	9	10
6	9	7	10	8
10	8	3	4	9
4	8	7	10	9
5	8	7	10	9
4	8	3	10	9
6	8	3	10	9
4	10	9	8	7
2	4	3	9	10
6	9	7	10	8
10	8	3	4	9
6	8	3	10	9
4	10	9	8	7
6	8	3	10	9
4	10	9	8	7
2	9	10	5	6
1	8	10	6	4
2	8	7	9	10
2	10	7	9	8
3	8	7	10	4
1	10	8	9	7

1	7	8	10	6
2	9	6	10	8
1	9	8	10	7
5	6	8	9	10
3	9	7	10	8
1	3	2	4	10
6	8	3	10	9
4	10	9	8	7
2	9	10	5	6
2	10	7	9	8
3	8	7	10	4
1	10	8	9	7
3	8	7	10	4
1	8	7	10	9
7	9	6	8	3
4	10	9	6	2
5	8	4	9	6
2	9	4	7	3
3	8	5	10	6
3	10	4	8	9
3	8	4	10	5
3	8	4	10	5
9	8	10	4	3
7	9	6	8	3
4	10	9	6	2
2	6	5	4	3
1	8	7	10	9
7	10	4	8	5
4	7	5	10	6
7	9	6	8	3
4	10	9	6	2
5	8	4	9	6
3	9	6	8	4
3	7	5	6	8
2	10	9	5	4
7	8	2	9	10
2	7	6	8	9
7	6	5	8	9
7	9	3	8	10
10	4	6	5	7
7	8	4	5	6
3	6	5	7	8
1	10	8	9	7
2	9	10	5	6
6	8	9	7	10
2	10	9	5	4
7	8	2	9	10

8	9	4	5	10
2	9	10	5	6
5	6	8	7	3
4	10	9	8	7
7	10	9	5	2
9	7	8	6	10
8	9	7	2	6
3	10	8	4	5
4	9	10	3	2
10	4	7	5	6
6	3	4	9	10
4	3	6	7	10
6	9	2	1	5
8	9	5	6	7
4	2	9	8	10
7	9	6	8	3
8	7	6	5	9
4	10	9	6	2
5	7	6	8	9
7	8	2	9	10
4	6	5	9	10
4	9	10	3	2
6	9	7	3	8
3	10	8	4	5
4	9	10	3	2
4	10	9	2	3
2	10	9	5	4
7	9	10	8	5
1	9	2	3	10
4	10	5	6	7
4	10	9	5	2
1	10	2	8	7
7	9	10	8	5
1	7	6	8	9
7	8	10	9	4
2	10	3	9	4
7	9	10	8	5
4	9	5	2	8
7	8	10	9	3
1	9	2	3	10
3	10	7	9	8
4	7	5	10	9
6	7	8	10	9
3	10	4	8	9
4	10	9	5	2
4	8	3	7	2
5	9	8	7	3

7	8	10	9	4
1	10	2	8	7
10	9	8	4	3
7	8	10	9	4
6	7	10	9	4
1	10	2	8	7
4	8	7	10	9
6	10	9	4	2
10	9	8	7	4
7	9	10	8	5
7	8	10	9	3
4	10	9	6	2
6	7	8	10	9
7	9	10	8	5
1	9	2	3	10
10	9	8	7	4
7	9	10	8	5
3	9	10	6	4
7	9	10	8	5
10	9	8	7	4
1	10	2	8	7
7	9	10	8	5
1	9	2	3	10
4	10	9	5	2
10	9	8	7	4
3	6	8	9	10
7	8	10	9	4
3	6	8	9	10
4	10	9	5	2
3	10	7	2	9
4	8	7	10	9
7	9	6	3	8
1	7	6	8	9
3	10	2	7	9
10	9	8	7	4
3	9	10	6	4
4	10	9	5	2
3	10	7	9	8
3	6	8	9	10
7	9	6	3	8
4	10	9	6	2
7	10	9	8	6
6	10	9	4	2
4	10	9	8	3
7	9	8	6	10
2	10	7	8	9
7	6	8	9	10

7	10	9	8	6
3	10	9	8	7
4	10	9	8	3
1	10	9	8	7
7	10	9	8	6
6	9	10	8	7
6	10	9	4	2
2	10	7	9	8
4	10	9	6	2
7	6	8	10	9
2	10	7	8	9
7	6	8	10	9
2	8	7	10	9
4	10	9	8	3
7	10	9	8	6
3	9	8	10	2
4	10	9	8	3
8	10	5	6	4
7	9	3	10	5
7	10	5	8	2
7	10	9	8	6
4	10	6	9	7
2	10	7	8	9
7	9	3	8	4
4	10	9	8	3
8	9	4	7	3
3	10	5	9	6
7	10	5	9	3
7	10	9	8	6
6	7	9	10	8
4	10	9	8	3
4	10	8	7	9
5	4	7	9	10
4	9	10	8	3
4	10	9	8	3
4	10	9	7	3
7	10	9	8	6
2	4	5	10	7
4	9	10	8	3
8	9	10	4	5
4	10	9	7	3
2	4	5	7	8
4	9	10	8	3
8	9	10	5	7
4	10	9	7	3
9	5	10	6	7
4	9	10	8	3

5	9	10	7	2
2	4	5	10	7
6	8	7	9	10
9	10	4	5	7
4	10	5	2	7
5	7	8	10	9
5	10	9	8	4
5	10	9	8	4
3	9	10	7	5
5	10	9	8	4
5	8	6	9	7
3	9	10	7	5
4	6	7	10	8
5	10	9	8	4
3	7	10	5	6
3	9	10	7	5
4	9	7	8	10
5	10	9	8	4
3	7	4	6	5
9	10	4	5	3
4	9	5	10	6
8	9	5	10	2
7	9	3	8	4
3	7	10	9	8
3	8	9	10	7
3	8	2	9	1
6	8	9	7	5
5	10	9	8	4
5	10	9	8	4
5	10	9	7	8
7	8	9	4	1
5	10	9	8	4
5	6	7	9	8
5	10	9	8	4
4	5	8	9	6
7	10	4	9	5
7	8	9	4	1
5	10	9	8	4
8	10	5	6	7
4	5	8	9	6
5	10	9	8	4
5	8	7	10	9
4	5	8	9	6
5	10	9	8	4
7	8	9	4	1
5	10	9	8	4
7	8	9	4	1

9	10	4	5	3
4	5	8	9	6
3	7	4	6	5
5	10	9	8	4
1	10	2	8	3
7	8	9	4	1
6	3	2	7	1
6	8	9	7	5
5	4	3	9	8
7	8	9	4	1
5	10	9	8	4
9	6	2	7	3
5	10	9	8	4
3	7	4	6	5
6	8	9	7	5
7	9	10	3	2
7	8	9	4	1
10	4	2	8	9
4	5	8	9	6
7	10	9	8	1
10	6	7	3	8
7	8	9	4	6
3	8	7	9	10
6	7	8	10	9
10	6	7	3	8
6	8	7	10	9
6	5	4	9	10
6	8	7	10	9
3	8	7	9	10
4	8	7	10	9
10	6	7	3	8
8	10	7	6	9
3	8	4	9	10
7	10	9	8	2
2	7	8	9	10
8	10	9	6	5
10	6	7	3	8
5	7	8	9	10
6	10	7	9	8
3	8	7	9	10
7	10	9	8	1
2	5	7	8	6
6	10	7	9	8
2	6	7	9	8
6	9	8	10	7
4	8	7	9	2
10	7	6	5	9

6	5	4	9	10
7	10	9	8	1
4	7	8	10	9
3	8	7	9	10
6	5	4	9	10
6	8	7	10	9
4	7	6	9	5
6	10	7	9	8
2	6	5	7	8
6	5	4	9	10
2	7	8	9	10
7	10	9	8	1
10	6	7	3	8
6	5	4	9	10
10	7	6	5	9
4	5	8	9	6
2	9	7	8	10
6	10	7	9	8
3	9	7	10	8
7	8	9	4	6
3	8	7	10	9
2	5	7	8	6
3	9	8	10	7
6	8	7	10	9
6	9	8	10	7
6	5	4	9	10
6	10	7	9	8
2	9	7	8	10
3	8	4	9	10
2	4	8	10	9
3	9	7	10	8
6	10	7	9	8
