# Supplementary material for: Selection criteria and husbandry practices of indigenous chicken producers in Northwest Ethiopia
Source: Heliyon. 2024 Aug 10;10(16):e36094. doi: 10.1016/j.heliyon.2024.e36094 (PMC11366869; doi:10.1016/j.heliyon.2024.e36094)
Supplement: Multimedia component 5 [file mmc5.pdf]

PA	Agro-ecology	GROWTH RATE	PLUMAGE COLOUR	APPEARANCE	COMB TYPE
1	1	4	1	3	5
1	1	2	1	3	4
1	1	3	5	2	1
1	1	4	3	2	1
1	1	6	5	1	2
1	1	7	3	2	1
1	1	4	3	1	2
1	1	4	2	1	3
1	1	4	3	1	2
1	1	5	3	1	2
1	1	3	6	2	1
1	1	3	4	1	2
1	1	2	1	3	4
1	1	3	5	2	1
1	1	4	3	2	1
1	1	6	5	1	2
1	1	4	3	1	2
1	1	4	2	1	3
1	1	3	4	1	2
1	1	2	1	3	4
2	1	1	2	3	4
2	1	1	3	4	5
2	1	1	2	3	5
2	1	1	3	4	5
2	1	1	6	3	2
2	1	2	4	1	3
2	1	1	2	3	4
2	1	1	5	3	2
2	1	1	4	5	3
2	1	1	3	4	5
2	1	1	2	3	5
2	1	1	3	4	5
2	1	1	2	3	4
2	1	2	4	1	3
2	1	1	2	3	4
2	1	1	5	3	2
2	1	1	3	4	5
2	1	1	2	3	4
2	1	1	3	4	5
2	1	1	2	3	4
3	1	4	3	1	2
3	1	4	2	3	1
3	1	2	4	3	1
3	1	1	2	3	4
3	1	1	2	3	4
3	1	1	3	2	4
3	1	1	2	3	4
3	1	1	3	4	2
3	1	1	4	2	3

3	1	3	1	4	2
3	1	1	2	3	4
3	1	1	3	2	6
3	1	1	3	4	5
3	1	1	2	3	4
3	1	4	3	1	2
3	1	1	2	3	4
3	1	1	2	3	4
3	1	1	3	2	4
3	1	1	2	3	4
3	1	1	3	2	4
4	1	4	1	2	3
4	1	1	4	2	3
4	1	3	2	1	4
4	1	2	5	1	6
4	1	5	2	1	3
4	1	4	1	2	3
4	1	2	3	1	4
4	1	1	4	2	3
4	1	2	3	1	4
4	1	4	1	2	3
4	1	1	4	2	3
4	1	3	4	2	1
4	1	1	3	2	4
4	1	2	3	1	4
4	1	1	4	2	3
4	1	4	1	2	3
4	1	1	4	2	3
4	1	3	2	1	4
4	1	2	4	1	3
4	1	1	2	3	4
5	1	2	1	4	3
5	1	2	3	1	4
5	1	2	1	3	4
5	1	4	2	1	3
5	1	2	3	1	4
5	1	2	3	1	7
5	1	4	3	1	2
5	1	4	3	1	2
5	1	1	3	2	4
5	1	4	3	1	2
5	1	2	4	1	3
5	1	2	1	4	3
5	1	2	3	1	4
5	1	3	2	1	4
5	1	4	3	1	2
5	1	1	3	2	5
5	1	1	2	3	4
5	1	3	1	4	2
5	1	1	3	2	6

5	1	2	7	1	6
6	1	5	1	2	3
6	1	1	2	5	3
6	1	1	2	6	3
6	1	2	1	3	4
6	1	4	1	3	6
6	1	6	2	3	1
6	1	7	3	2	1
6	1	3	2	4	1
6	1	4	1	2	3
6	1	1	2	3	6
6	1	1	4	2	3
6	1	4	2	3	1
6	1	2	3	1	4
6	1	5	1	6	2
6	1	1	2	5	3
6	1	2	3	4	1
6	1	5	1	2	3
6	1	1	2	5	3
6	1	3	1	2	4
6	1	2	1	4	3
7	2	5	1	2	3
7	2	2	1	3	4
7	2	5	6	4	1
7	2	1	2	5	3
7	2	4	5	1	3
7	2	5	1	2	3
7	2	4	7	1	2
7	2	3	4	7	1
7	2	1	5	4	2
7	2	5	1	2	3
7	2	5	3	1	2
7	2	5	6	3	4
7	2	2	1	3	4
7	2	4	1	2	3
7	2	2	1	3	4
7	2	3	2	1	5
7	2	1	3	2	4
7	2	1	2	5	3
7	2	5	6	1	3
7	2	2	1	4	3
8	2	3	4	7	1
8	2	4	5	1	3
8	2	3	4	5	2
8	2	3	4	7	1
8	2	6	7	3	4
8	2	4	5	1	3
8	2	3	4	2	1
8	2	1	2	3	4
8	2	3	4	5	6

8	2	5	1	2	3
8	2	5	6	3	4
8	2	4	1	3	2
8	2	3	2	1	5
8	2	5	1	2	3
8	2	2	1	3	4
8	2	3	4	5	6
8	2	5	1	2	3
8	2	3	6	2	1
8	2	5	1	2	3
8	2	3	4	5	6
9	2	4	5	1	3
9	2	5	1	2	3
9	2	2	1	3	4
9	2	1	2	5	3
9	2	3	4	5	6
9	2	3	4	2	1
9	2	3	4	7	1
9	2	3	4	2	1
9	2	1	2	5	3
9	2	3	4	2	1
9	2	3	4	2	1
9	2	3	4	2	1
9	2	4	7	1	2
9	2	3	4	2	1
9	2	3	4	5	6
9	2	3	6	2	1
9	2	1	2	5	3
9	2	4	1	2	3
9	2	4	1	2	5
9	2	3	4	2	1
10	2	4	1	3	2
10	2	1	3	4	2
10	2	1	2	3	4
10	2	2	4	3	1
10	2	1	2	4	3
10	2	4	3	2	1
10	2	1	2	3	4
10	2	1	3	4	2
10	2	4	3	2	1
10	2	2	4	3	1
10	2	4	3	2	1
10	2	1	3	4	2
10	2	2	4	3	1
10	2	1	2	3	4
10	2	4	3	2	1
10	2	4	1	3	2
10	2	1	2	4	3
10	2	4	3	2	1
10	2	1	2	4	3

10	2	4	3	2	1
11	2	2	4	3	1
11	2	1	3	4	2
11	2	3	2	4	1
11	2	2	4	3	1
11	2	3	1	6	2
11	2	2	3	5	1
11	2	1	2	4	3
11	2	1	3	4	2
11	2	3	2	6	1
11	2	4	3	2	1
11	2	2	5	6	1
11	2	2	4	3	1
11	2	4	2	3	1
11	2	4	5	1	3
11	2	3	1	5	6
11	2	1	3	4	2
11	2	1	5	2	4
11	2	2	4	3	1
11	2	2	1	4	3
11	2	3	2	4	1
12	2	3	1	4	2
12	2	2	4	3	1
12	2	1	2	4	3
12	2	1	3	4	2
12	2	4	1	6	7
12	2	3	1	4	2
12	2	4	2	1	6
12	2	1	2	4	3
12	2	4	1	6	3
12	2	3	1	4	2
12	2	4	1	2	6
12	2	1	2	4	3
12	2	4	6	1	2
12	2	3	1	4	2
12	2	1	3	6	2
12	2	4	1	6	7
12	2	2	4	3	6
12	2	5	1	6	2
12	2	4	1	6	2
12	2	2	4	3	5
13	3	4	3	2	1
13	3	4	3	2	1
13	3	4	1	2	3
13	3	4	3	2	1
13	3	3	2	1	4
13	3	4	1	2	3
13	3	7	1	2	3
13	3	4	3	2	1
13	3	4	2	3	1

13	3	4	1	2	3
13	3	3	1	2	4
13	3	4	3	2	1
13	3	4	1	3	2
13	3	2	3	1	7
13	3	2	1	5	3
13	3	4	1	2	3
13	3	2	3	4	1
13	3	3	4	1	2
13	3	4	3	2	1
13	3	3	1	2	4
14	3	1	2	3	5
14	3	4	3	2	1
14	3	4	3	2	1
14	3	3	1	4	2
14	3	2	1	3	4
14	3	4	3	2	1
14	3	1	2	5	6
14	3	4	3	2	1
14	3	4	3	1	2
14	3	1	2	5	4
14	3	2	1	3	4
14	3	4	3	2	1
14	3	1	2	5	4
14	3	4	3	1	2
14	3	4	3	2	1
14	3	1	2	5	4
14	3	4	3	1	2
14	3	4	3	2	1
14	3	2	1	3	4
14	3	4	3	2	1
15	3	2	1	3	4
15	3	2	3	1	7
15	3	4	3	1	2
15	3	4	1	3	2
15	3	4	3	2	1
15	3	1	7	5	2
15	3	2	1	3	4
15	3	4	5	7	1
15	3	1	2	3	5
15	3	4	7	5	6
15	3	2	1	3	4
15	3	4	3	2	1
15	3	1	3	4	5
15	3	4	3	2	1
15	3	4	1	3	2
15	3	1	2	3	5
15	3	7	6	4	5
15	3	2	1	3	4
15	3	1	2	6	7

15	3	4	3	1	2
16	3	5	1	3	2
16	3	2	4	3	1
16	3	1	2	3	5
16	3	1	4	3	2
16	3	1	2	3	4
16	3	2	4	3	1
16	3	2	1	3	4
16	3	3	1	2	4
16	3	2	3	1	4
16	3	1	4	3	2
16	3	1	3	2	4
16	3	2	4	3	1
16	3	4	1	2	3
16	3	4	1	2	3
16	3	1	2	3	4
16	3	3	2	1	4
16	3	1	2	3	5
16	3	2	4	3	1
16	3	1	2	6	3
16	3	4	1	3	2
17	3	1	4	3	2
17	3	5	1	3	2
17	3	2	1	3	4
17	3	5	1	2	3
17	3	4	1	3	2
17	3	3	1	2	4
17	3	2	1	3	4
17	3	4	1	3	2
17	3	3	2	1	4
17	3	5	1	3	2
17	3	2	1	3	4
17	3	1	4	3	2
17	3	3	1	2	4
17	3	2	3	1	4
17	3	3	2	4	1
17	3	5	1	2	3
17	3	1	2	3	4
17	3	3	1	2	4
17	3	3	2	1	4
17	3	5	1	3	2
18	3	2	4	3	1
18	3	3	1	2	4
18	3	4	1	3	2
18	3	4	3	1	2
18	3	5	1	2	3
18	3	5	1	2	3
18	3	1	2	3	4
18	3	1	2	3	5
18	3	5	1	2	3

18	3	2	1	3	4
18	3	4	1	2	3
18	3	2	3	1	4
18	3	3	1	2	4
18	3	3	1	2	4
18	3	4	1	3	2
18	3	6	1	2	3
18	3	4	1	2	3
18	3	4	1	2	3
18	3	4	1	3	2
18	3	5	1	2	3

SCAVENGING ABILITY	DISEASE RESISTANCE	LONGEVITY
6	7	2
6	7	5
7	4	6
5	6	7
7	3	4
4	5	6
5	6	7
7	5	6
5	6	7
6	4	7
5	4	7
5	6	7
6	7	5
7	4	6
5	6	7
7	3	4
5	6	7
7	5	6
5	6	7
6	7	5
7	6	5
6	2	7
6	4	7
6	2	7
5	4	7
6	5	7
6	5	7
6	4	7
6	2	7
6	2	7
6	4	7
6	2	7
7	6	5
6	5	7
6	5	7
6	4	7
6	2	7
7	6	5
6	2	7
7	6	5
6	7	5
7	6	5
6	5	7
6	5	7
6	5	7
5	6	7
6	5	7
6	5	7
6	5	7

5	6	7
6	5	7
7	5	4
6	2	7
7	6	5
6	7	5
6	5	7
6	5	7
5	6	7
6	5	7
5	6	7
6	7	5
7	5	6
6	5	7
3	4	7
6	4	7
7	5	6
6	5	7
6	5	7
7	5	6
6	7	5
7	5	6
7	5	6
5	6	7
6	5	7
5	6	7
6	7	5
7	5	6
6	5	7
6	5	2
7	6	5
7	6	5
6	5	7
7	6	5
6	5	7
6	5	7
4	6	5
6	5	7
6	5	7
5	6	7
6	7	5
6	7	5
7	6	5
6	5	7
7	6	5
6	7	5
4	6	7
7	6	5
6	7	5
4	5	7

5	3	4
7	6	4
7	6	4
5	4	7
6	5	7
2	5	7
4	5	7
5	4	6
5	6	7
6	7	5
4	5	7
7	5	6
6	5	7
6	5	7
3	4	7
7	6	4
7	5	6
7	6	4
7	6	4
7	6	5
7	6	5
6	7	4
7	5	6
7	2	3
7	6	4
7	2	6
6	7	4
6	3	5
2	5	6
6	3	7
6	7	4
6	4	7
3	2	1
7	5	6
7	6	5
7	5	6
4	6	7
7	5	6
7	6	4
7	2	4
5	6	7
2	5	6
7	2	6
1	7	6
2	5	6
1	2	5
7	2	6
6	5	7
7	6	5
7	2	1

6	7	4
3	2	1
7	6	5
4	6	7
6	7	4
7	5	6
7	2	1
6	7	4
5	7	4
6	7	4
7	2	1
7	2	6
6	7	4
7	5	6
7	6	4
7	2	1
5	7	6
2	5	6
5	7	6
7	6	4
5	7	6
6	5	7
5	7	8
6	3	5
5	7	6
7	2	1
5	7	4
7	6	4
7	6	5
3	7	6
5	7	6
7	6	5
5	7	6
7	6	5
5	7	6
6	7	5
5	6	7
6	7	5
5	7	6
7	6	5
5	7	6
5	7	6
5	7	6
7	6	5
7	6	5
5	6	7
7	6	5
6	7	5
5	6	7
6	7	5

6	5	7
5	7	6
5	7	6
7	6	5
5	7	6
7	4	5
7	4	6
6	5	7
5	7	6
5	4	7
5	6	7
7	4	3
5	7	6
6	7	5
2	7	6
7	4	2
5	7	6
3	6	7
5	7	6
5	7	6
6	7	5
5	7	6
5	7	6
6	5	7
5	7	6
3	2	5
5	7	6
7	5	3
6	5	7
7	4	7
5	7	6
5	3	7
6	5	7
7	3	5
5	7	6
5	7	4
2	3	5
5	7	1
7	3	4
3	7	5
6	1	7
7	6	5
7	6	5
7	6	5
7	6	5
5	6	7
7	6	5
4	5	6
7	6	5
8	7	6

7	6	5
5	6	7
7	6	5
5	7	6
6	4	5
6	4	7
6	5	7
7	5	6
5	7	6
7	5	6
6	5	7
7	4	6
7	6	5
7	6	5
6	7	5
5	6	7
7	6	5
4	3	7
7	6	5
6	7	5
7	3	6
5	6	7
7	6	5
7	3	6
6	7	5
7	6	5
7	3	6
6	7	5
7	6	5
5	6	7
7	6	5
5	6	7
6	4	5
6	7	5
5	7	6
7	6	5
4	3	6
5	6	7
3	2	6
7	4	6
3	2	1
5	6	7
7	6	5
6	2	7
7	6	5
5	7	6
7	4	6
3	2	1
5	6	7
3	4	5

6	7	5
6	7	4
7	5	6
6	7	4
6	5	7
5	7	6
7	5	6
5	6	7
5	6	7
5	6	7
6	5	7
6	5	7
7	5	6
6	5	7
7	5	6
7	6	5
6	5	7
6	7	4
7	5	6
5	4	7
6	5	7
6	5	7
6	7	4
5	6	7
7	4	6
6	5	7
5	6	7
5	6	7
6	5	7
6	5	7
6	7	4
7	5	6
6	5	7
5	6	7
5	6	7
5	6	7
7	4	6
6	5	7
5	6	7
6	5	7
6	7	4
7	5	6
5	6	7
6	5	7
6	7	5
6	4	7
7	4	6
6	5	7
6	7	4
6	4	7

5	6	7
6	5	7
5	6	7
5	6	7
5	6	7
6	5	7
5	4	7
7	5	6
6	5	7
6	5	7
7	4	6
