# Supplementary material for: Selection criteria and husbandry practices of indigenous chicken producers in Northwest Ethiopia
Source: Heliyon. 2024 Aug 10;10(16):e36094. doi: 10.1016/j.heliyon.2024.e36094 (PMC11366869; doi:10.1016/j.heliyon.2024.e36094)
Supplement: Multimedia component 6 [file mmc6.docx]

**Section 1: Questionnaire**

**General information and socio economic aspects survey**

1. **Zone**: ____________**Woreda**: ____________**Kebele**: ____________ **HH No**: ______________
2. GPS reference for PAs **Longitude**: __________**Latitude**: _________**Altitude**: _____________
3. **Enumerator**: ______________________**Interviewee**: ________________**Date**:_____________

| **4. Sex of the respondent*(****encircle one)* | | | | | | | | 5. **5. Age of the respondent**_________ | | |
| --- | --- | --- | --- | --- | --- | --- | --- | --- | --- | --- |
| 1. Male | | | | | | | | **6. Education level** (*encircle one*) | | |
| 2. Female | | | | | | | | 1. Illiterate | | |
| **7. Position of the respondent in the household *(****encircle one)* | | | | | | | | 2. Write and read | | |
| 1. Household head | | | | | | | | 3. Grade 10^th^ and 12^th^ completed | | |
| 2. Spouse of head | | | | | | | | 4. College graduated | | |
| 3. Relative  4. Son | | | | | | | | **9. What is your major farming activity?*(****encircle one)* | | |
| 5. Daughter | | | | | | | | 1. Livestock production | | |
| **8. Marital status** (*encircle one*) | | | | | | | | 1. Crop production | | |
| 1. Married | | | | | | | | 1. Both | | |
| 2. Divorced  3. Widowed | | | | | | | | **10. On which do you depend more for?** (*encircle one*) | | |
| **11. Numbers of livestock keeping by the household** | | | | | | | | 1. Food(home consumption) | | |
| **Livestock** | **Number** | | **Livestock** | | **Number** | |  | 1. Income source | | |
| Chickens |  | | Donkey | |  | |  | **12. Breed/ecotype of your chicken ( may be local name):___________** | | |
| Cattle |  | | Mule | |  | |  |  |  |  |
| Sheep |  | | Horse | |  | |  | **13. Special attribute/s of your chickens?** ____________________ | | |
| Goat |  | |  | |  | |  |  |  |  |
| **14. Chicken flock composition** | | | | | | | |  |  |  |
| **Chicken types** | | **Number** | | | | | |  |  |  |
|  | | Local | | Exotic | |  | |  |  |  |
| 1. Hens (Laying) | |  | |  | |  | |  |  |  |
| 1. Cocks (Mature) | |  | |  | |  | |  |  |  |
| 1. Pullets | |  | |  | |  | |  |  |  |
| 1. Cockerels | |  | |  | |  | |  |  |  |
| 1. Chicks | |  | |  | |  | |  |  |  |

| **15. Population trend in major livestock species for the last ten years** *(Tick one box and write the reason)* | | | | | | | | |
| --- | --- | --- | --- | --- | --- | --- | --- | --- |
| livestock species | Increasing | | Decreasing | | Stable | | Reason | |
| 1. Chickens |  |  | |  |  |  |  |  |
| 1. Sheep |  |  |  |  |  |  |  |  |
| 1. Cattle |  |  |  |  |  |  |  |  |
| 1. Goat |  |  |  |  |  |  |  |  |

**Production and management system aspect survey**

1. **General**
   1. **Main purpose of keeping chickens (***Rank based on their purposes***)**

| **Male chickens** | |  | **Female chickens** | |
| --- | --- | --- | --- | --- |
| **Purpose** | ***Rank*** |  | **Purpose** | ***Rank*** |
| 1. Meat (home consumption) |  |  | 1. Eggs (home consumption) |  |
| 1. Source of income (sale) |  |  | 2. Eggs (for sale) |  |
| 1. Manure |  |  | 3. Source of income (sale) |  |
| 1. Breeding |  |  | 4. Meat (home consumption) |  |
| 1. Savings |  |  | 5. Manure |  |
| 1. Wealth Status |  |  | 6. Breeding |  |
| 1. Dowry |  |  | 7. Savings |  |
| 1. Ceremonies |  |  | 8. Wealth Status |  |
| Others (*Specify*) |  |  | 9. Dowry |  |
| _______________ |  |  | 10. Ceremonies |  |
|  |  |  | Others (*Specify*)  _______________ |  |

|  | **1.2. Members of household responsible in chicken rearing activities**  *(Tick one or more boxes in each column and row;* (M = Male, F= Female) | | | | | | |
| --- | --- | --- | --- | --- | --- | --- | --- |
|  | | (≤18y) | |  | (>18y) | | |
|  | | M | F |  | M | F |  |
| 1. Purchasing of chickens | |  |  |  |  |  |  |
| 2. Selling of chickens | |  |  |  |  |  |  |
| 3. Selling of eggs | |  |  |  |  |  |  |
| 4. Caring for sick animals | |  |  |  |  |  |  |
| 5. Feeding | |  |  |  |  |  |  |
| 6. Cleaning of perches and houses | |  |  |  |  |  |  |
|  | Others (*specify*)__________ | | | | | | |

**2. Feeding, Watering and Housing**

**2.1.** What type of management system do you practice for your chicken rising? (*encircle one*)

1. Extensive 2. Semi-intensive 3. Intensive 4. Other, specify____________

**2.2.** What nutritional management do you apply for your chickens? (*encircle one*)

1. Scavenging 2. Scavenging + Supplement 3. Confined, complete ration

**2.3.** What are the feed resources for your chickens?__________________________________

__________________________________________________________________________

**2.4.** Do you give water for your birds? 1. Yes 2. No

**2.5.** I f yes, where do you get water from? (*encircle one or more*) 1. Borehole 2. Rain water 3. River 4. Well 5. Tap water 6. Others, specify _________________

**2.6.** Where do your birds rest at night? (*encircle one*)

1. In the family house 2. Separate shelter 3. Separate house with other animals

4. Others, specify___________________Tree

1. **Health and disease control**

| **3.1.** List of diseases which occur frequently and affect the productivity of chickens in the area  ( *Rank them based on importance*) | | | | | | |
| --- | --- | --- | --- | --- | --- | --- |
| Type of disease | Symptom | Season of occurrence | Susceptible age group | Rank | Treatment | |
|  |  |  |  |  | Modern | Traditional |
|  |  |  |  |  |  |  |
|  |  |  |  |  |  |  |
|  |  |  |  |  |  |  |
|  |  |  |  |  |  |  |
|  |  |  |  |  |  |  |

**3.2.** Do you give vaccination for your chickens? 1. Yes 2. No

| Type of vaccination | Sex | Age | Season |
| --- | --- | --- | --- |
|  |  |  |  |
|  |  |  |  |

- 1. If your answer is **yes**,

1. **Breeding practices**
   1. Do you practice selection for breeding cocks? 1. Yes 2. No
   2. Do you practice selection for breeding hens? 1. Yes 2. No

**4.3.** Selection criteria for breeding hens and cocks

| **Breeding hens** | | |  | **Breeding cocks** | | | |
| --- | --- | --- | --- | --- | --- | --- | --- |
| No | Traits | *Rank* |  | *No* | Traits | | *Rank* |
| 1. | Egg numbers |  |  | 1 | Growth rate/weight | |  |
| 2. | Broodiness |  |  | 2 | Plumage color | |  |
| 3. | Fertility of eggs |  |  | 3 | “*Qumena*” | |  |
| 4. | Plumage color |  |  | 4 | Comb type | |  |
| 5 | “*Qumena*” |  |  | 5 | Scavenging ability | |  |
| 6 | Comb type |  |  | 6 | Disease resistance | |  |
| 7 | Scavenging ability |  |  | 7 | Longevity | |  |
| 8 | Disease resistance |  |  | Others (*Specify*) _____________ | | | |
| 9 | Longevity |  |  |  |  |  | |
| 10 | Mothering ability |  |  |  |  |  | |
| Others (*Specify*) _____________ | | |  |  |  |  | |

- 1. Incubation method (*encircle one*)

1. Natural (Broody hen) 2. Artificial incubation 3. Both

- 1. Breeding/mating system(*encircle one*)

1. Uncontrolled (natural) 2. Controlled

- 1. Broody behavior modification (*encircle one*)

1. Nothing 2. Hanging upside-down 3. Moving to neighbor houses

| - 1. Trait categories/factors most influencing price of live chickens | | **4.8.** Farmers’ preferences for plumage colours and comb types | | |
| --- | --- | --- | --- | --- |
| **Trait category/ Factor** | ***Rank*** |  | **Traits** | ***Rank*** |
| 1. Plumage colour |  |  | **Plumage colour** |  |
| 1. Weight/size |  |  | White |  |
| 1. Comb type |  |  | Red |  |
| 1. Shank colour |  |  | Black |  |
| 1. Breed/ecotype |  |  | *Gebesima* |  |
| Others (*specify*) ________ |  |  | *Ambesima* |  |
|  |  |  | *Teterima* |  |
|  |  |  | Any colour |  |
|  |  |  | **Comb type** |  |
|  |  |  | Single (“*Netella*”) |  |
|  |  |  | Double (“*Dirib*”) |  |
|  |  |  | Any type |  |

4. Submerge into water up to the breast 5. Change brooding place 6. Others, *specify*_______ **4.9.** How do you select productive hens for egg production? (*Rank*)

| **Selection method** | **Rank** |
| --- | --- |
| 1. Body size |  |
| 1. By finger accommodation between the pelvic bones |  |
| 1. By pedigree performance for replacement |  |
| 1. By using comp types |  |
| Others, *specify* ______________ |  |

- 1. Do you have your own cock? 1. Yes 2. No
  2. If yes, where is the source of your cock? 1. Own (private flock) 2. Purchased (Market)
  3. If no, where do you get cock for your hens?

1. From neighbor 2. I do not need a cock for my hens 3. Others, *specify* _____________
   1. What is the purpose of keeping breeding cock?
2. For mating 2. Socio-cultural purpose 3. For meat 4. Others, *specify* __________

**4.14.** Do you practice culling? 1. Yes 2. No

**4.15.** For what purpose/s do you cull chickens? (*Tick one or more boxes)*

| **Purpose of culling** | **Tick** |
| --- | --- |
| 1. for consumption |  |
| 1. for sale |  |
| 1. for sacrifice |  |
| Others, *specify ______________* |  |

- 1. What are the criteria’s to cull chickens? (R*ank*)

| **Criteria’s to cull** | **Rank** |  | - 1. At what age of the bird do you decide to cull? (*Tick on one box*) | |
| --- | --- | --- | --- | --- |
| 1. Poor productivity |  |  | **Culling age** | **Tick** |
| 2. Old age |  |  | 1. >3 years |  |
| 3. Sickness |  |  | 2. >4years |  |
| 4. Lack of broodiness  Others, *specify ___________* |  |  | 3. >5years |  |
|  |  |  | 4. Birds do not cull based on their age |  |
|  |  |  | Others, s*pecify _____________* |  |

**Thank you!**

|  |  |  |  |  |
| --- | --- | --- | --- | --- |
